# Time‐restricted feeding prior to *Mycobacterium tuberculosis* infection reduces tissue CD4+ T cells with limited impact on bacterial clearance

**DOI:** 10.1002/2211-5463.70263

**Published:** 2026-06-04

**Authors:** Ashish Gupta, Nidhi Yadav, Subhasmita Das, R. Rajendra Kumar Reddy, Nupur Sharma, Amol Ratnakar Suryawanshi, Jaswinder Singh Maras, Ranjan Kumar Nanda

**Affiliations:** ^1^ Translational Health Group International Centre for Genetic Engineering and Biotechnology New Delhi India; ^2^ Clinical Proteomics Laboratory BRIC‐Institute of Life Sciences Bhubaneswar India; ^3^ ILS Mass Spectrometry Facility BRIC‐Institute of Life Sciences Bhubaneswar India; ^4^ Department of Molecular and Cellular Medicine Institute of Liver and Biliary Sciences New Delhi India

**Keywords:** immunology, metabolomics, *Mycobacterium tuberculosis*, time‐restricted feeding

## Abstract

The effects of time‐restricted feeding (TRF) on immune responses during bacterial infection are not well‐studied. Here, we subjected mice (6–8 weeks, male) to 8 h of TRF for 30 days and then infected them with a low dose of *Mycobacterium tuberculosis* (Mtb) H37Rv. During the first 15 days, TRF improved glucose tolerance with marginal weight loss. However, global serum and liver metabolomics alongside liver proteomics indicated that TRF perturbed fatty acid biosynthesis and degradation, steroid hormone biosynthesis, and tyrosine metabolism. Together, these results indicate that TRF potentially affected the distribution and functionality of host immune cells. TRF mice had similar mycobacterial burdens in lungs and spleen at 21 days postinfection but had significantly lower CD3^+^ T cells in bone marrow and CD4^+^ T cells in both bone marrow and lungs. Ultimately, we show that TRF induced changes in amino acid and lipid metabolism persist during Mtb infection.

## Graphical abstract

Time‐restricted feeding (TRF) in mice increased liver fatty acid oxidation and decreased fatty acid biosynthesis. These alterations persisted when TRF was discontinued and the host was infected with *Mycobacterium tuberculosis.* Pre‐exposure to TRF did not alter tissue (lung and spleen) mycobacterial burden but significantly reduced CD3^+^ T cells in lungs and CD3^+^ and CD4^+^ T cells in bone marrow.

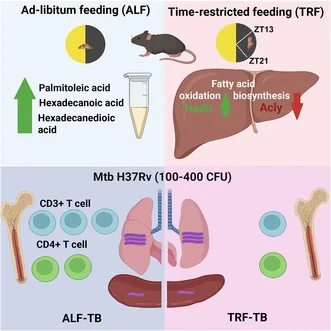

**Abbreviations**
ACN: acetonitrile
ALF: *ad libitum* feeding
BSL: biosafety level
CFU: colony forming unit
CR: caloric restriction
DPI: days post infection
ELISA: enzyme‐linked immunosorbent assay
FA: formic acid
FDR: false discovery rate
GC–MS: gas chromatography mass spectrometry
HFD: high‐fat diet
HOMA‐IR: homeostatic model assessment of insulin resistance
ICGEB: International Centre for Genetic Engineering and Biotechnology
iPGTT: intraperitoneal glucose tolerance test
LC–MS: liquid chromatography and mass spectrometry
MCT: microcentrifuge tube
MSTFA: N‐methyl‐*n*‐(trimethylsilyl)trifluoroacetamide
Mtb: *Mycobacterium tuberculosis*
PCA: principal component analysis
SEM: standard error of mean
TB: tuberculosis
TCEP: Tris(2‐carboxyethyl)phosphine
TEAB: Triethylammonium bicarbonate
TRE: time‐restricted eating
TRF: time‐restricted feeding
UHPLC: ultra‐high‐performance liquid chromatography
ZT: zeitgeber

Time‐restricted eating or feeding (TRE/F) is a dietary intervention that restricts the time window of food intake to 6–12 h a day in humans or animal models. This intervention extends the daily fasting window, reducing body weight and improving metabolic status in overweight, obese, and prediabetic individuals [1, 2]. However, limited studies have investigated the impact of TRF on the immune system and whether it resolves or worsens infectious disease conditions such as tuberculosis (TB) is unknown.

TRF improves the host metabolic parameters by synchronizing food availability with the expression of enzymes involved in nutrient utilization [2]. Night‐restricted TRF in mice, which limits food access to the lights‐off phase, is reported to reduce fat mass, dampen high‐fat diet‐induced weight increase, and improve glucose homeostasis [2, 3, 4, 5]. TRF in mice is also reported to reduce inflammation by lowering the expression of inflammatory cytokines and chemokines, but limited reports have investigated its impact in the context of infection [2, 6, 7, 8, 9, 10, 11].

In this study, we aimed to investigate the effect of 30 days of TRF on the immune system of male C57BL/6 mice and its impact on *Mycobacterium tuberculosis* (Mtb) H37Rv infection. TRF appears to enhance host metabolism at the tissue or cellular level, and establishing its role in resolving infections may aid in developing targeted dietary interventions that benefit TB patients.

## Experimental procedures

### Study animals

All experiments in this study were performed in accordance with the approved procedures of the Institutional Animal Ethics Committee of the International Centre for Genetic Engineering and Biotechnology, New Delhi (ICGEB/IAEC/16072024/41.8). Male C57BL/6 mice (6–8 weeks) were used in this study. Five animals per cage were housed and kept under a 12‐h light/dark cycle, with lights on at 8:00 AM (ZT0) and off at 8:00 PM (ZT12). The cages had appropriate nesting material. Mice groups were randomly assigned to either *ad libitum* (ALF) or a time‐restricted feeding (TRF) group and fed a normal chow diet (65% carbohydrates, 24% protein, 11% fat sourced from Altromin, Lage, Germany; Cat no. 1324). Mice in the TRF group had access to food only for 8 h from Zeitgeber time (ZT) 13–ZT21, where ZT0 is the time when the lights are switched on, and ZT12 is the time when the lights are switched off. Throughout the entire experimental duration, food intake and body weight of the mice were measured daily and weekly, respectively.

### Intraperitoneal glucose tolerance test (iPGTT)

After fasting the mice for 16 h (ZT21 to ZT13), glucose (1 g·kg^−1^ body weight) was introduced intraperitoneally and blood glucose levels were measured from the tail vein at different time intervals (0, 15, 30, 60, 90, and 120 min) using a glucometer (Dr. Morepen BG‐03 Gluco One Glucometer). At the indicated time points, whole blood samples were collected via retro‐orbital bleeding from the mice (after 16 h of fasting) and, after 20 min, were centrifuged at 3500 ***g*** for 20 min at 4 °C to separate the serum. The serum was then aliquoted and stored at −80 °C for further analysis.

### Serum insulin and free fatty acid measurement

Insulin levels were measured in the serum of mice after 16 h of fasting, using the Rat/Mouse insulin ELISA kit (Cat no. EZRMI‐13K; Millipore Sigma, MO, USA) following the manufacturer's instructions. And homeostatic model assessment of insulin resistance (HOMA‐IR) was calculated using the following formula: [12]

$$\;\text{HOMA}-\mathrm{IR}=\frac{\text{Insulin}\left(\mathrm{\mu IU}/\mathrm{mL}\right)\times \text{Fasting glucose}\left(\text{mmol}/L\right)}{22.5}$$

Fasting‐free fatty acid levels in the serum of experimental mouse groups were measured using a fluorometric assay (Cat no. 700310; Cayman Chemical, MI, USA) following the manufacturer's instructions.

### Serum and liver tissue metabolite isolation and mass spectrometry analysis

The tissue samples were collected between ZT0 (i.e., after the end of the feeding/active period) and ZT2. To the serum samples (50 μL), chilled methanol (400 μL) and internal standard (ribitol, 2 μL, 0.5 mg·mL^−1^) were added, and after vortexing, the samples were incubated at −20 °C overnight. These processed serum samples were centrifuged at 16 000 ***g*** for 10 min at 4 °C, and the supernatant was dried in a SpeedVac (Labconco, MO, USA). The dried pellet was resuspended in 5% acetonitrile and transferred to an autosampler vial for global metabolite profiling by using liquid chromatography and mass spectrometry (LC–MS). The LC–MS data were acquired using a Thermo Scientific™ UHPLC system combined with a Q Exactive Orbitrap Mass Spectrometer. Hypersil GOLD™ C18 Selectivity HPLC Column was used for metabolite separation. The mobile phases consisted of 1% formic acid as solvent A and 100% acetonitrile. The gradient was 1 min, 5% solvent B; 17 min, 99% solvent B; 21 min, 99% solvent B; 22 min, 5% solvent B; 25 min, 5% solvent B. The solvent flow rate was 0.5 mL·min^−1^, and the column temperature was 60 °C. The ion source temperature ranged from 250 to 300 °C, with a spray voltage of 5500–4500 V. The MS data were acquired in both positive and negative modes, with a mass range of 150 to 2000 Da, using nitrogen as the collision gas, and at a resolution of 70 000 m·δm^−1^. The LC–MS data were processed using Compound Discoverer 3.3 to identify the metabolites.

Similarly, to the liver (50 mg) tissue, chilled methanol (400 μL, 80%), internal standard (ribitol, 2 μL, 0.5 mg·mL^−1^), and zirconium beads (2 mm, 250 mg) were added and homogenized in 3 cycles (30 s on; 30 s off) using a bead beater (Biospec Mini‐Beadbeater‐16). After bead beating, the extracted liver metabolites were incubated on ice for 30 min. Following incubation, the samples were centrifuged at 16 000 ***g*** for 10 min at 4 °C, and the supernatant was dried using a SpeedVac (Labconco, MO, USA). To these dried, processed samples, methoxamine hydrochloride (20 mg·mL^−1^, 40 μL) was added, and the mixture was incubated at 60 °C for 2 h at 900 rpm in a thermomixer. To the reaction mixture, N‐methyl‐*n*‐(trimethylsilyl)trifluoroacetamide (MSTFA, 70 μL) was added, and the mixture was incubated at 60°C for 30 min at 900 rpm. After incubation, the samples were centrifuged at 10 000 ***g*** for 10 min at 25 °C, and the supernatant was transferred to gas chromatography (GC) vial inserts for data acquisition using a 7890 Gas Chromatograph coupled to a Pegasus 4D GC × GC‐time‐of‐flight mass spectrometer. The derivatized samples (1 μL) were injected into an HP‐5 ms column (30 m length, 0.25 mm width, 250 μm internal diameter) in splitless mode using helium as a carrier gas at a constant flow rate (1 mL·min^−1^). The secondary column was Rxi‐17 (1.5 m length and 250 μm diameter). Electron ionization mode was fixed at −70 eV to scan ions of 33 to 600 m/z range at an acquisition rate of 20 spectra/s. The ion source temperature was set at 220 °C. The GC oven parameters used for acquisition were as follows: 50 °C hold for 1 min, followed by temperature increase to 200 °C at 8.5 °C·min^−1^, then to 280 °C at 6 °C·min^−1^, with a hold time of 5 min. The secondary oven temperature offset was set at 5 °C relative to the GC oven temperature, and the modulator temperature offset was set at 15 °C relative to the secondary oven temperature. The transfer line temperature was set at 225 °C. A solvent delay of 600 s was used during data acquisition, and GC–MS data of derivatized samples were acquired within 24 h of derivatization. All GC–MS raw data files of the study groups were aligned using the ‘Statistical Compare’ feature of chromatof (4.50.8.0; Leco Corporation, MI, USA), and metadata were prepared.

### Liver protein isolation and processing for proteomics experiment

Frozen liver tissue (100 mg) was sliced into thin pieces and transferred to a bead‐beating tube containing 5 zirconia beads (2 mm) and 500 μL lysis buffer (20 mm HEPES, 100 mm NaCl, 0.05% Triton X‐100, 1 mm DTT). Bead beating was carried out in an MP Biomedical Bead beating homogenizer with 20 s on/1 min off twice. The tissue lysate was clarified by centrifuging at 12 000 ***g*** for 15 min at 4 °C, and the supernatant was then recentrifuged at 12 000 ***g*** for 10 min at 4 °C. The amount of protein in the tissue lysates was quantified using the Bicinchoninic acid assay method.

An equal amount of liver protein (100 μg) from each biological replicate was dried in a SpeedVac (Labconco). After resuspending it in TEAB (100 mm, 100 μL), the proteins were reduced by incubating with TCEP (200 mm, 5 μL) for 1 h at 55 °C. Then, these proteins were alkylated by incubating with iodoacetamide (375 mm, 5 μL) at room temperature for 30 min in the dark. These proteins were precipitated using prechilled acetone (6 : 1, v/v) and by incubating overnight at −20 °C. These mixtures were centrifuged at 8000 ***g*** for 10 min at 4 °C. The pellet was resuspended in TEAB (100 mm, 100 μL) and incubated overnight with sequencing‐grade trypsin (2.5 μg) at 37 °C. Digestion was stopped by the addition of 41 μL anhydrous acetonitrile, and the tryptic peptides were dried in a SpeedVac (Labconco) at 40 °C. The dried peptides were resuspended in 300 μL of 0.1% trifluoroacetic acid and desalted using Pierce™ Peptide Desalting Spin Columns by following the manufacturer's instructions. The eluted peptides were dried in a SpeedVac at 40 °C and stored for LC–MS/MS data acquisition.

### Liver proteomics data acquisition using LC–MS/MS and analysis

The dried tryptic peptides were resuspended in 0.1% Formic Acid (Cat no. 5330020050; Merck, MA, USA) in MS‐grade water (Cat no. AAB‐W6‐4; Fisher Scientific). One μg of tryptic peptides was loaded and separated first on PepMap Neo C18 5 μm 300 μm × 5 mm 1500 bar Trap column (Cat no. 174500; Thermo Scientific, NJ, USA) and then on EASY‐Spray PepMap Neo C18 Column, 2 μm, 75 μm × 500 mm, 1500 bar (Cat no. ES75500PN; Thermo Scientific) using Thermo Scientific Vanquish Neo UHPLC system at a flow rate of 300 nL·min^−1^ using a gradient of solvent A (100% H_2_0 + 0.1% FA) and solvent B (80% ACN + 0.1% FA). The peptides were separated by reversed‐phase liquid chromatography using a gradient from 5% to 50% solvent B over 120 min at a flow rate of 300 nL·min^−1^. Further, the MS1 data were acquired in positive‐ion mode using an Orbitrap with a resolution of 60 000 and a mass range of 350 to 1200 m/z. Precursor ions were fragmented using higher‐energy C‐trap dissociation (HCD) in an ion trap (IT) with a collision energy of 30% in a data‐dependent mode, accompanied by a rapid IT scan rate. Precursor ions with +2 to +7 charge and monoisotopic ions were selected. Parent ions, once fragmented, were excluded for 60 s with an exclusion mass width of ±10 ppm.

The raw MS/MS data were searched against the UniProt proteome database of *Mus musculus* (UP000000589; 54 690 sequences on 2025_06_06) at an FDR of 5% using proteome discoverer software version 3.1.0.638 (Thermo Scientific). A maximum of two trypsin cleavages was allowed with a precursor mass tolerance of 10 ppm and fragment mass tolerance of 0.6 Da. Carbamidomethylation (+ 57.021 Da) of cysteine residues at the C terminus was selected as the static modification, and oxidation of methionine residues (+ 15.995 Da) and acetylation at the N terminus (+ 42.011 Da) were selected as the dynamic modifications. Peptides and master proteins identified with high confidence were annotated, and their quantitative abundances were normalized to the same total peptide amount. The final matrix was used for statistical analysis, and proteins with a log_2_ fold change ≥ ±1.0 and a −log_10_ adjusted *P*‐value of ≥ 1.3 were classified as deregulated.

### Mtb H37Rv infection

A set of mice completing 30 days of TRF and a control ALF group were aerosol infected with a low dose (100–120 colony‐forming units; CFU) of animal‐passaged Mtb H37Rv using a Madison chamber in the tuberculosis aerosol challenge facility (BSL‐III) in ICGEB. Mice were humanely euthanized on Days 1 and 21 post‐Mtb H37Rv infection to monitor their tissue mycobacterial burden. During this period, mice infected with Mtb from both the TRF and ALF groups had access to food for 24 h. Mice were humanely euthanized following the approved institutional guidelines by cervical dislocation under isoflurane anesthesia. Tissues (lung, spleen, and liver) were aseptically collected and homogenized in phosphate‐buffered saline, and the tissue homogenates were plated on 7H11 agar plates supplemented with PANTA. The plates were incubated at 37 °C in a humidified incubator for 21 days, and the colonies were enumerated to calculate the CFU.

### Tissue immune cell phenotyping

Harvested tissues (lungs, spleen) of experimental mouse groups were incubated with collagenase D (1 μg·mL^−1^) and DNase I (0.5 mg·mL^−1^) in RPMI 1640 medium at 37 °C for 30 min. The cell suspension was passed through a cell strainer (70 μm) and centrifuged at 400 ***g*** at 4 °C for 5 min. The cell pellet was lysed with RBC cell lysis buffer at room temperature for 5 min. For immune cell isolation from bone marrow, the epiphyses of the femur and tibia were cut with scissors and placed in a 0.6 mL microcentrifuge tube (MCT), then kept in a separate 2 mL MCT. After centrifuging at 400 ***g*** for 5 s, the cell pellet was collected and then resuspended in RPMI 1640 medium. The cells were centrifuged, and the pellet was resuspended in RBC cell lysis buffer and incubated for 5 min at room temperature.

Immune cells from (lungs, spleen, and bone marrow) were washed with PBS before staining with viability dye (LIVE/DEAD™ Fixable Near‐IR Dead Cell Stain) and surface markers (CD3‐FITC; Cat no. 100203 and CD4‐BV785; Cat no. 100453 from BioLegend, CA, USA) on ice for 30 min. After washing, the cells were resuspended in FACS buffer (0.5% FBS in PBS) before data acquisition using BD LSRFortessa X‐20, and data were analyzed using fcs express Version 6.0.

### Cytokine estimation

Serum cytokine levels were measured using the LEGENDplex Mix and Match mouse inflammation panel following the manufacturer's instructions (BioLegend). The data were acquired using a BD LSRFortessa X‐20 and analyzed using the web‐based LEGENDplex™ Data Analysis Software Suite.

### Data analysis

All group‐specific data were presented as mean ± standard error of the mean (sem). The Mann–Whitney or Welch's *t*‐test was used to assess statistical significance in parameters between the two groups, and a *P*‐value < 0.05 at a 95% confidence interval was considered statistically significant. The iPGTT data were analyzed using two‐way analysis of variance (ANOVA). The final data matrix generated from metabolomics and proteomics data were uploaded to metaboanalyst (6.0), and the data were log‐transformed and auto‐scaled for multivariate data analysis. Metabolites or proteins with a log_2_ fold change of ≥ ±1.0 and a log_10_‐adjusted *P*‐value ≥ 1.3 were classified as deregulated molecules. The plots were generated using graphpad prism Version 8 or the R package ‘ggplot2’.

## Results

### TRF influences body weight gain and impacts host glucose homeostasis

To monitor the effect of TRF on the host, 6–8 weeks C57BL/6 mice were subjected to a TRF regimen with access to food for 8 h from ZT13 to ZT21, whereas the control *ad libitum* (ALF) group had unrestricted access to food (Fig. 1A). The TRF group consumed a similar amount of food to the ALF group (Fig. 1B) and, in the initial 2 weeks, lost 1.5% of body weight (Fig. 1C). After 30 days of TRF, the mice gained 5.49% of body weight, whereas the ALF control group gained 16.62% of body weight compared to the baseline (Fig. 1C). At Days 7 and 14, mice undergoing TRF lost significant body weight compared to the *ad libitum* controls (Fig. S1A).

**Fig. 1:**
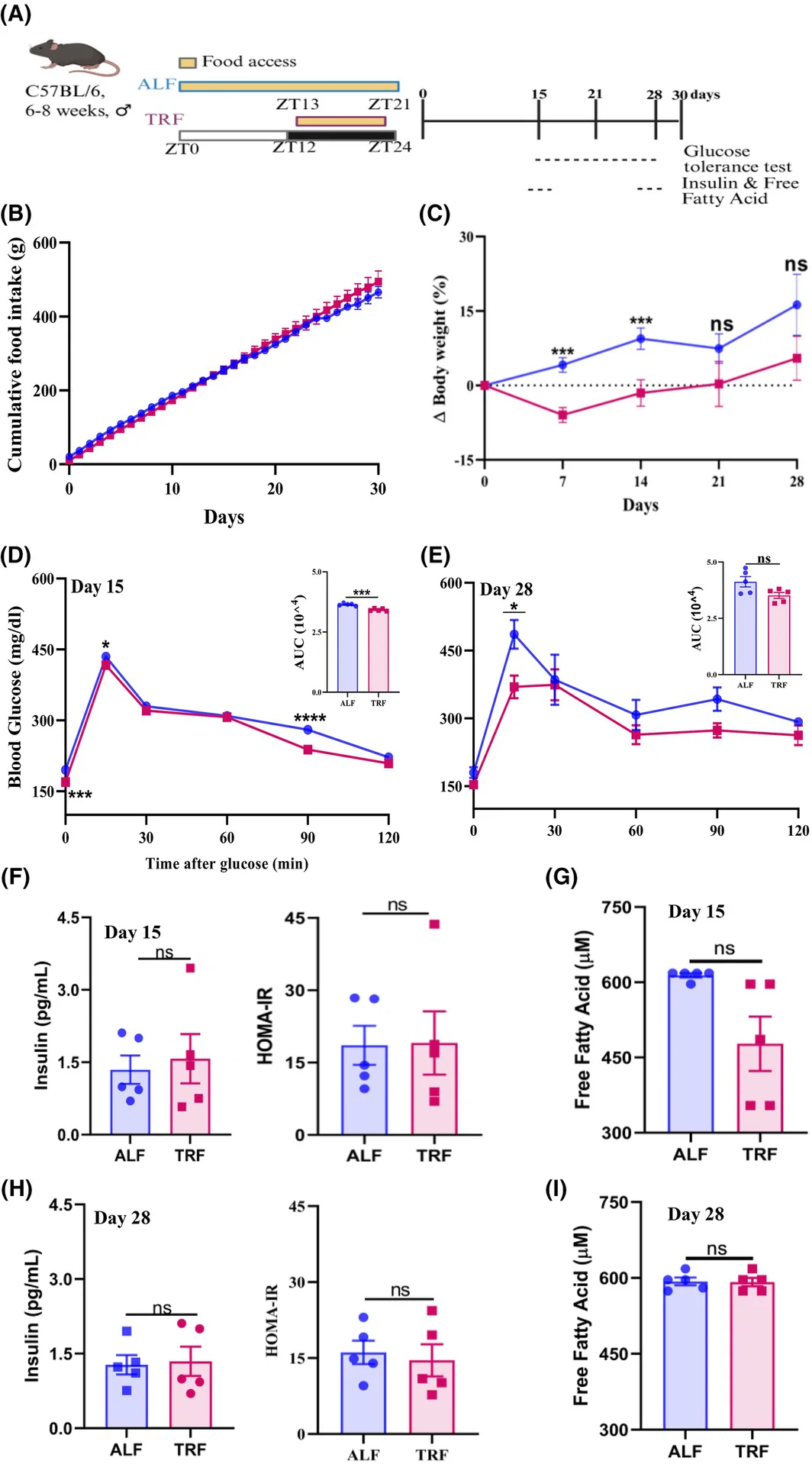
Time‐restricted feeding (TRF) alters the glucose homeostasis at early point but does not affect fasting serum insulin and free fatty acid levels. (A) Schematic of the experimental design adopted in the study. (B) Cumulative food intake per cage in grams. (C) Change in body weight with time. (D, E) Intraperitoneal glucose tolerance test at Days 15 and 28 post‐TRF. The quantification of the area under the curve (AUC) above baseline is shown in the inserts. (F, H) Insulin levels after 16 h of fasting and corresponding homeostatic model assessment of insulin resistance (HOMA‐IR) levels at Day 15 and Day 28, respectively. (G, I) Free fatty acid levels after 16 h of fasting at Day 15 and Day 28, respectively. The number of mice analyzed per group for each figure was 5, except for (B), for which 2 cages per group were analyzed. Each dot represents a biological replicate. Statistical significance was determined using the Holm–Sidak method with correction for multiple comparisons in (B, C), two‐way repeated‐measures ANOVA and Sidak's multiple comparisons tests for ipGTT and unpaired *t*‐test with Welch's correction for AUC in (D, E) and unpaired *t*‐test with Welch's correction in (F–I). The error bar represents the standard error of the mean. *P ≤ 0.05, ***P ≤ 0.001, ****P ≤ 0.0001, ns = not significant. Created in biorender. Yadav, N. (2026) https://BioRender.com/uheibho.

On Day 14, the TRF group had a significantly lower area under the curve for the intraperitoneal glucose tolerance test (Fig. 1D). At Days 21 and 28, both the TRF and ALF groups had similar areas under the curve for the intraperitoneal glucose tolerance test, indicating similar glucose tolerance (Fig. 1E and Fig. S1B). Fasting serum insulin and free fatty acid levels and HOMA‐IR between the TRF and ALF mice were similar at Days 15 and 28 (Fig. 1F–I). Initiation of TRF resulted in significant weight loss at initial time points (up to Day 14), but at later time points, body weight was similar between the ALF and TRF groups. Similar observations, such as minimal body weight gain and changes in glucose homeostasis, have been reported in humans undergoing TRE [1].

### TRF alters amino acid and fatty acid metabolism

We performed a global metabolomics experiment to monitor TRF‐induced metabolic alterations in the circulation and liver, if any (Fig. 2A). We selected serum to monitor systemic changes, and the liver, due to its critical contribution as a metabolic hub. Principal component analysis (PCA) of the serum metabolome showed overlap between the TRF and ALF groups (Fig. 2B). A set of 1123 metabolites was identified in the serum, out of which 51 were deregulated (log_2_ fold change ≥ ±1.0; −log_10_ *P*‐value > 1.3) (Table S1). These deregulated molecules contributed to the metabolism of amino acids (alanine, aspartate, glutamate, arginine, proline, and tyrosine), lipid biosynthesis, including the elongation and degradation of fatty acids, pyrimidine metabolism, and steroid hormone biosynthesis pathways (Fig. 2C,D). Serum palmitoleic acid levels were significantly low, and higher cytidine and cystine levels were observed in the TRF groups, indicating an altered fatty acid and pyrimidine metabolism between the study groups (Fig. S2A). Similarly, global liver metabolome data showed slight overlap in the PCA plot (Fig. S2B,C). Through GC–MS‐based metabolite analysis, we captured 119 liver metabolites, out of which eight (propanoic acid, methylmaleic acid, gluconic acid, lysine, benzaldehyde, 10‐undecynoic acid, arabinonic acid, and ribonolactone) were dysregulated (log_2_ fold change ≥ ±1.0; *P*‐value < 0.05) in the TRF mice (Table S2). These molecules are involved in biotin, propanoate metabolism, the pentose phosphate pathway, and lysine degradation pathways, indicating significant differences in liver metabolic phenotypes between the TRF and ALF groups (Fig. S2D,E).

**Fig. 2:**
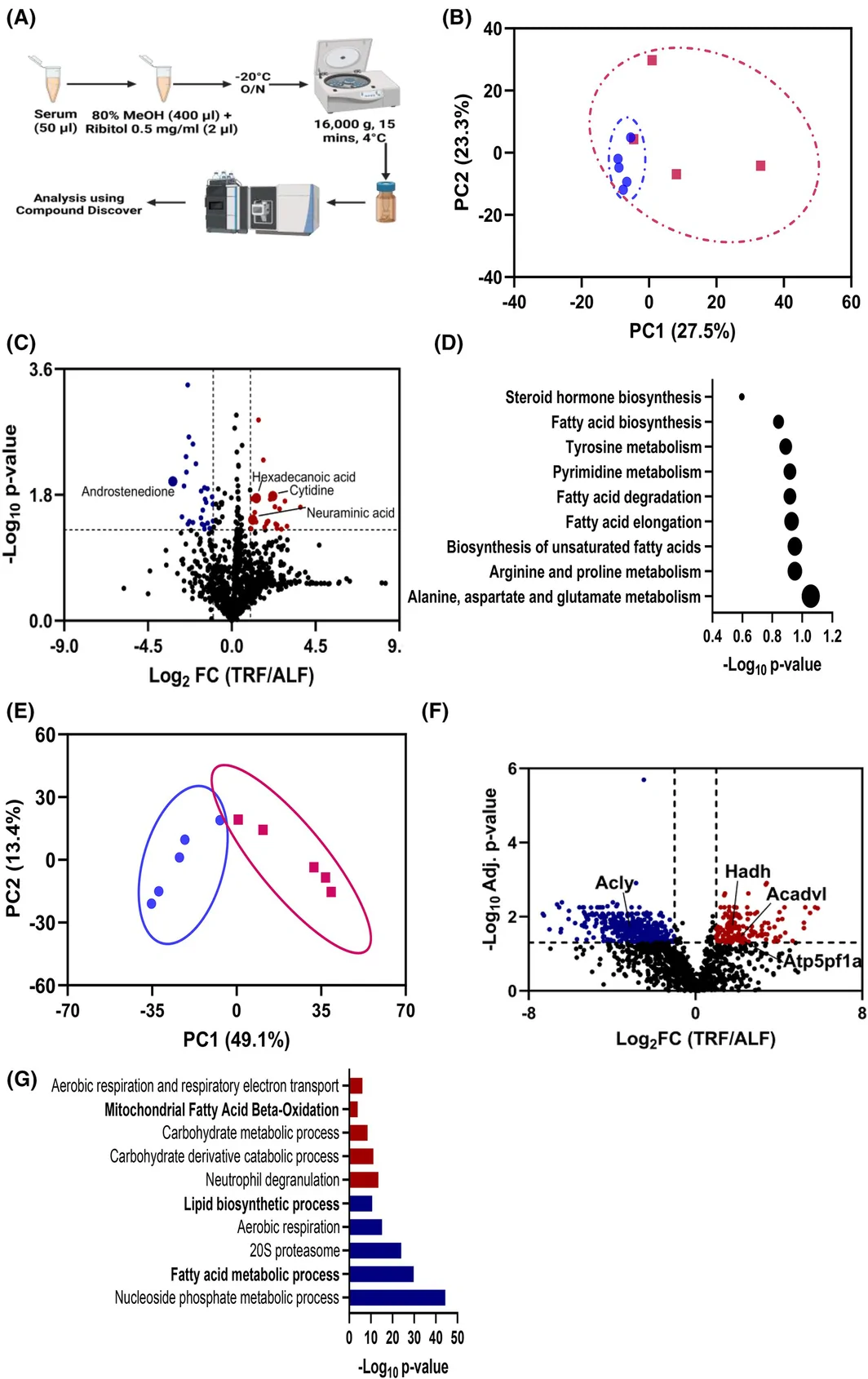
Time‐restricted feeding (TRF)‐induced remodeling of metabolism was observed at both tissue and systemic circulation levels. (A) Schematic representation of the method used for metabolite profiling from the TRF and *ad libitum* feeding (ALF) mice serum using liquid chromatography mass spectrometry. (B) Principal component analysis (PCA) of the serum metabolites between the TRF and ALF mice. (C) Volcano plot showing the deregulated metabolites in the serum of the TRF and ALF mice. (D) Metabolite set enrichment analysis (MSEA) of the deregulated metabolites shown in (C). The dot size represents the enrichment ratio. (E) PCA of the liver proteomics between the TRF and ALF mice. (F) Volcano plot showing the deregulated proteins in the liver of the TRF and ALF mice. Adj. stands for adjusted. (G) Pathway enrichment analysis of the deregulated proteins shown in (F). Blue circle and burgundy square represent the ALF and TRF group, respectively, in the PCA plots in (B, E). Upregulated metabolites/proteins/pathways are shown in red, whereas the deregulated metabolites/proteins/pathways are shown in blue in (C, F, G). Created in biorender. Yadav, N. (2026) https://BioRender.com/uheibho.

TRF also majorly impacted the liver proteome and showed a clear separation between the ALF and TRF mice (Fig. 2E). TRF mice liver showed decreased abundance of 437 proteins (log_2_ fold change ≥ −1.0; *P*‐value < 0.05), and a set of 152 proteins had higher abundance (log_2_ fold change ≥ 1.0; *P*‐value < 0.05; Fig. 2F, Table S3). The pathway enrichment analysis using the deregulated liver proteome data showed upregulation of fatty acid beta‐oxidation and downregulation of lipid biosynthetic process (Fig. 2G). Apart from the increased utilization of fatty acids, TRF mice had higher abundance of proteins such as ATP synthase F (1) complex subunit alpha, cytochrome c oxidase subunit 5A, and lgyceraldehyde‐3‐phosphate dehydrogenase involved in aerobic respiration and the electron transport chain. We performed a global correlation analysis on the significantly deregulated liver metabolites and the top 20 up‐ and downregulated proteins. We observed a significant positive correlation between methylmaleic acid, propanoic acid and lysine, with Hao1 involved in fatty acid alpha‐oxidation (Fig. S2F). Thus, 30 days of continuous 8‐h TRF in mice resulted in significant alterations in amino and fatty acid metabolism at both systemic and tissue levels.

### Pre‐exposure to TRF for 30 days minimally affected tissue mycobacterial burden at 21 days postinfection (dpi)

To determine whether 30 days of TRF improved the host's ability to handle respiratory infections such as Mtb, C57BL/6 mice completing 30 days of TRF were aerosol infected with 100–400 CFU of animal passaged Mtb H37Rv (Fig. 3A and Fig. S3A). After Mtb infection, the TRF mice group received food *ad libitum*, like the ALF group. The Mtb‐infected TRF and ALF control mice had similar body weight at 21 dpi (Fig. 3B and Fig. S3B). At 21 dpi, random blood glucose levels between the Mtb‐infected TRF and ALF mice were similar (Fig. S3C). The gross tissue pathology of the TRF‐TB and ALF‐TB mouse groups showed similar pathology (Fig. 3C). At 21 dpi, the Mtb‐infected TRF and ALF mice groups had similar mycobacterial burdens in their lungs and spleens (Fig. 3D). Therefore, pre‐exposure to TRF for 30 days did not benefit the C57BL/6 mice in terms of tissue mycobacterial clearance at the early time point, that is, 21 dpi. However, TRF mice infected with Mtb may have impacted the tissue immune system.

**Fig. 3:**
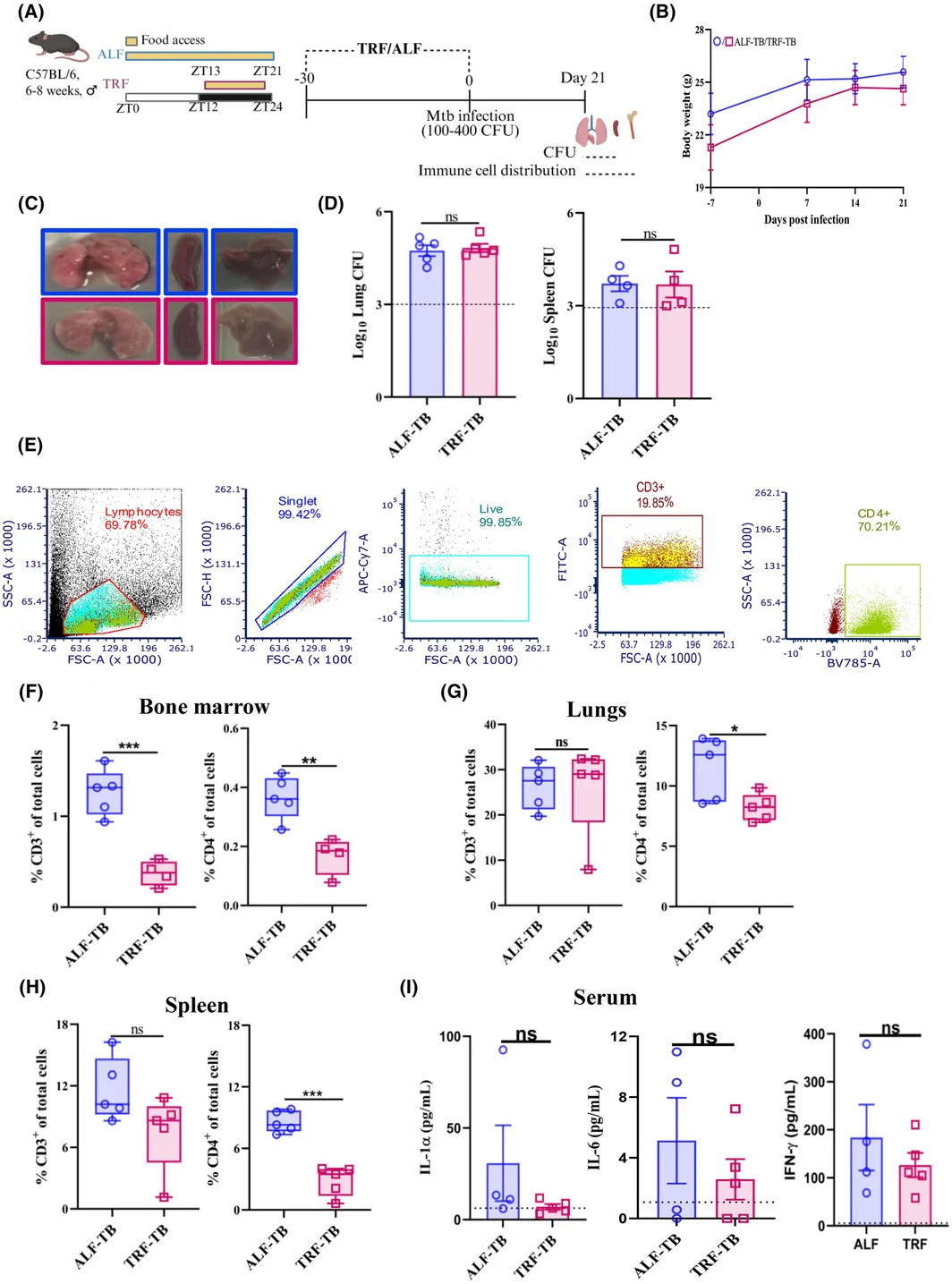
Time‐restricted feeding (TRF) decreases the CD4^+^ T cell number in a tissue‐dependent manner at 21 days post‐Mtb H37Rv infection (dpi). (A) Schematic of experimental design adopted in the study. (B) Change in body weight with time after *Mycobacterium tuberculosis* (Mtb) infection. (C) Gross pathology of the lungs, spleen and liver of the *ad libitum* feeding‐tuberculosis (ALF‐TB) and TRF‐TB mice. (D) Mycobacterial burden in the lungs and spleen at 21 dpi. (E) The gating strategy adopted to determine CD3^+^ T cells and CD3^+^ CD4^+^ T cells. Percentage of CD3^+^ and CD3^+^ CD4^+^ T cells in (F) bone marrow, (G) lungs and (H) spleen. (I) Levels of pro‐inflammatory cytokines IL‐1α, IFN‐γ and IL‐6 in the serum. Each dot represents a biological replicate. Statistical significance was determined using the Holm‐Sidak method with correction for multiple comparisons in (B) and unpaired *t*‐test with Welch's correction in (D, F–I). The error bar represents the standard error of the mean in (B, D, I); and the error bar represents the range of the data in (F–H). **P ≤ 0.01 and ***P ≤ 0.001. Created in biorender. Yadav, N. (2026) https://BioRender.com/uheibho.

### Mtb‐infected mice pre‐exposed to TRF showed differential tissue immune cell distribution

To monitor the tissue immune cell distribution of these Mtb‐infected TRF and control ALF mice groups, we monitored the distribution of CD3^+^ and CD4^+^ T cells in multiple tissues (bone marrow, lungs, and spleen) at 21 dpi. Gating strategy shown in Fig. 3E. The bone marrow of TRF‐TB mice had significantly lower CD3^+^ and CD4^+^ T cells compared to the ALF‐TB group (Fig. 3F and Fig. S4A). Similarly, the lung CD4^+^ T cell population was significantly lower in the TRF‐TB group (Fig. 3G and Fig. S4B). The absolute number of splenic CD3^+^ and CD4^+^ T cells were similar between the study groups with a significantly lower frequency of CD4^+^ T cells in the TRF‐TB mice (Fig. 3H and Fig. S4C). The circulatory IL‐6, IFN‐γ, and IL‐α levels between Mtb‐infected TRF and ALF mice groups at 21 dpi were similar (Fig. 3I). Thus, pre‐exposure to TRF decreased the number of key immune cell types such as CD3^+^ and CD4^+^ T cells required for host defense against Mtb, without adversely affecting tissue mycobacterial clearance at early time point, that is, 21 dpi.

### TRF‐induced metabolic changes persisted in TRF‐TB mice

To determine whether TRF‐induced metabolic alterations in circulation and the liver persist after TRF is discontinued, following Mtb infection, a global metabolomics experiment was conducted (Fig. 4A). The PCA plot of the serum metabolome data showed a clear separation between the TRF‐TB and ALF‐TB groups (Fig. 4B). Out of the identified 1120 serum metabolites, 22 were deregulated (log_2_ fold change ≥ ±1.0; *P*‐value < 0.05) in the TRF‐TB group (Table S4). Pathway enrichment analysis of the deregulated metabolites identified in serum revealed perturbed nicotinate and nicotinamide metabolism, sphingolipid metabolism, and fatty acid and steroid hormone biosynthesis (Fig. 4C,D). The PCA plot of the liver metabolome data of TRF‐TB and ALF‐TB showed a slight overlap (Fig. S5A). Out of the 121 identified metabolites in the liver, the TRF‐TB mice group showed 14 deregulated (log_2_ fold change ≥ ±1.0; *P*‐value < 0.05) metabolites (Fig. S5B and Table S5). Pathway enrichment analysis of deregulated metabolites revealed perturbations in linoleic acid metabolism, fructose and mannose metabolism, galactose metabolism, biosynthesis of unsaturated fatty acids, tyrosine metabolism, amino acid and nucleotide sugar metabolism, and arachidonic metabolism (Fig. S5B,C). Therefore, it is essential to note that TRF‐induced metabolic changes persisted in the TRF mice following Mtb infection.

**Fig. 4:**
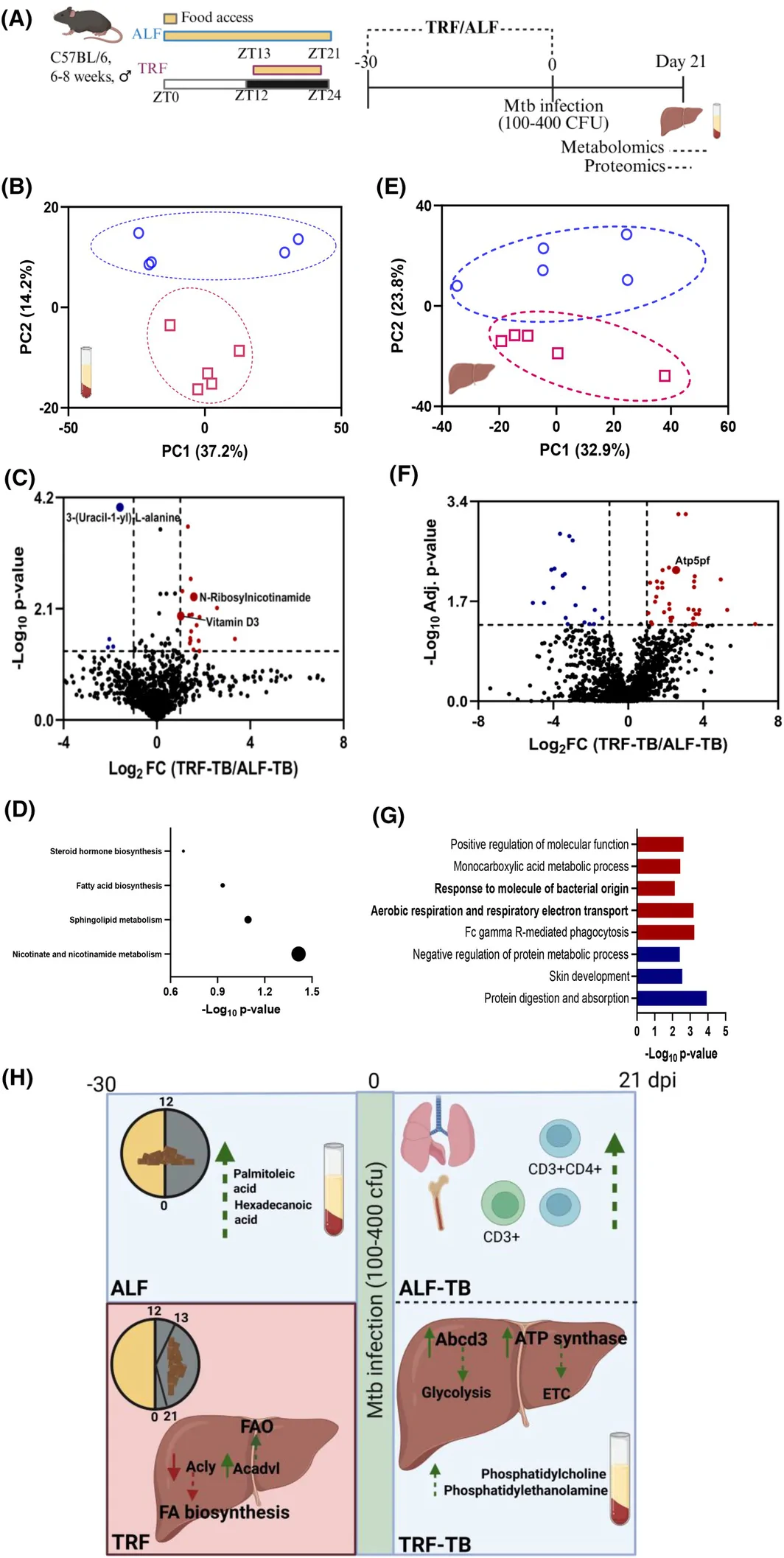
Time‐restricted feeding (TRF)‐induced metabolic changes persist post 21 days of Mtb infection. (A) Schematic of experimental design adopted in the study. (B) Principal component analysis (PCA) of the serum metabolites between the TRF‐TB (tuberculosis) and ALF‐TB mice. (C) Volcano plot showing the deregulated metabolites in the serum of the TRF‐TB and ALF‐TB mice. (D) Metabolite set enrichment analysis (MSEA) of the deregulated metabolites shown. The size of the dot represents the enrichment ratio. (E) PCA of liver proteome between the TRF‐TB and ALF‐TB mice. (F) Volcano plot showing the deregulated proteins in the liver of the TRF‐TB and ALF‐TB mice. Adj. stands for adjusted. (G) Pathway enrichment analysis of deregulated proteins shown in (F) Blue open circle and burgundy open square represent the ALF‐TB and TRF‐TB group, respectively in the PCA plots in (B, E) Upregulated metabolites/proteins/pathways are shown in red, whereas the deregulated metabolites/proteins/pathways are shown in blue in (C, F–H) Summary of TRF's effect on the host metabolism and the immune system. The left‐hand side represents the summary of the results prior to Mtb infection, whereas the right‐hand side represents the summary of the results after Mtb infection. The top half represents ALF or ALF‐TB, whereas the bottom half represents TRF or TRF‐TB. Levels of palmitoleic acid and hexadecenoic acid were significantly higher in the ALF mice serum. Fatty acid biosynthesis (FAO; Acly) related proteins were deregulated in the liver, whereas fatty acid oxidation (FA; Acadvl) related proteins were upregulated in the liver of TRF mice. CD3^+^ T cells (represented in green) and CD3^+^CD4^+^ T cells (represented in blue) were significantly higher in the bone marrow, and CD3^+^CD4^+^ T cells were significantly higher in the lungs of ALF mice. Glycolysis (Abcd3) and electron transport chain (ATP synthase) related proteins were upregulated in the liver of TRF‐TB mice. Created in biorender. Yadav, N. (2026) https://BioRender.com/uheibho.

Similar to metabolic analysis, we investigated whether TRF‐induced changes in the liver proteome persist after Mtb infection. The PCA plot showed clear difference in the liver proteome of the ALF‐TB and TRF‐TB mice (Fig. 4E). TRF‐TB mice had a set of 56 deregulated liver proteins (log_2_ fold change ≥ ±1.0; adjusted *P*‐value < 0.05; Fig. 4F and Table S6). The pathway enrichment analysis showed significant upregulation of metabolic pathways (aerobic respiration and respiratory electron chain, and monocarboxylic acid metabolic process) and immune system‐related pathways (response to molecules of bacterial origin and Fc gamma R‐mediated phagocytosis; Fig. 4G). We performed a global correlation analysis on the significantly deregulated liver metabolites and the top 20 downregulated/upregulated proteins in the ALF‐TB vs. TRF‐TB mice. We observed a significant positive correlation between proteins involved in the electron transport chain (Atp5f, Cox5a, Sdhaf4; Fig. S5D). Thus, alterations induced by TRF in amino acid and fatty acid metabolism persist even after its discontinuation.

## Discussion

In this study, we investigated the effect of TRF on the host immune system, specifically during infection with the intracellular pathogen *Mycobacterium tuberculosis*. TRF (or TRE) is a dietary intervention that reduces the food consumption window (a minimum of less than 12 h) without altering the quality or quantity of food consumed. This has been shown to have pleiotropic metabolic benefits, especially in the context of lifestyle‐induced metabolic diseases.

We observed that a 30‐day treatment with TRF in C57BL/6 male mice resulted in significant body weight loss during the initial period, which was not observed at later time points and subsequently returned to normal. That corroborates earlier reports that the effect of TRF on body weight was observed only at early time points. Multiple reports yielded mixed findings on the loss of body weight in humans of various ethnicities who adopted TRE for specified time periods. Most reports showed minimal body weight loss in humans undergoing TRE [1, 13, 14, 15]. Mice receiving an high‐fat diet (HFD) following TRF show significantly lower body weight gain compared than ALF controls [2, 16]. In our study, mice had access to a normal chow diet in which only 11% of calories were derived from fat, whereas in the HFD, fat is responsible for 60% of calories. We observed better glucose homeostasis in TRF mice at an early time (Day 15 of TRF), but the effect was abrogated by Day 28. During this period, we did not observe any differences in fasting insulin levels or HOMA‐IR between the TRF and ALF mice. Previous studies have reported pleiotropic effects of TRF on fasting blood glucose levels in mice receiving HFD, with some study showing a positive effect, whereas others show no significant improvement [8, 9, 17]. This could be due to lack of insulin resistance in 6‐ to 8‐week‐old male C57BL/6 mice being fed a normal chow diet, in contrast to those fed HFD.

Mice undergoing TRF experience continuous cycles of feeding and fasting, which has a profound impact on nutrient utilization, which can impact the immune system. It is well‐established that host metabolism profoundly impacts the host immune response against Mtb. We observed that TRF remodeled the host metabolome in the circulation and metabolically active tissue, such as the liver (Fig. 4H). Pathways related to amino acid metabolism (alanine, aspartate and glutamate metabolism, arginine and proline metabolism, and tyrosine metabolism), fatty acid metabolism (biosynthesis of unsaturated fatty acids, fatty acid elongation, fatty acid degradation, fatty acid biosynthesis, and steroid hormone biosynthesis), and pyrimidine metabolism were enriched in the circulation of mice completing 30 days of TRF. Pathways associated with biotin metabolism, propanoate metabolism, pentose phosphate pathway, and lysine degradation were enriched in the liver of TRF mice. The liver proteome profile of TRF mice indicated a perturbed fatty acid metabolism. The pathway enrichment analysis revealed an increased reliance on oxidative phosphorylation and fatty acid metabolism to meet the host's energetic requirements. Fatty acid and amino acid metabolism‐related pathways remain altered at 21 dpi even though TRF is discontinued prior to Mtb infection (Fig. 4H). Earlier reports have similarly demonstrated that TRF reduces fatty acid biosynthesis and increases fatty acid oxidation [2, 18, 19, 20]. Chaix *et al*., [2] reported that the ALF group mice serum had higher levels of metabolites involved in the sterol/steroid pathway. TRE in humans has been reported to influence pathways associated with amino acids and lipid metabolism [18]. Intermittent fasting has also been shown to alter lipid metabolism in mice fed a high‐fat diet [19]. Caloric restriction (CR) reported to alter liver metabolome resulting in upregulation of linoleic acid metabolism, and findings of our study corroborates the same [20]. TRF reported to upregulate proteins related to oxidative phosphorylation and electron transport chain in the inguinal white adipose and skeletal muscles and similar changes are reflected in the liver proteome profile reported in our study [21, 22]. Alternate day fasting is also reported to upregulate proteins involved in fatty acid oxidation in the liver [23]. Our study has demonstrated that this alteration in lipid metabolism induced by TRF is quite robust, as the effects persist even if TRF is discontinued and the host is infected with an intracellular pathogen, that is, Mtb [24]. We did not observe that the same metabolites or proteins were deregulated after 30 days of TRF and 21 dpi, but there was a commonality in the pathways that were altered at these two time points.

Very few studies have investigated the effects of TRF on the host immune system during bacterial infection. We assessed the effect of TRF on both the tissue mycobacterial burden and CD3^+^ and CD4^+^ T cells at 21 dpi, as the adaptive immune response peaks and the Mtb levels reach a plateau in C57BL/6 mice by 21 dpi [25]. The circulatory pro‐inflammatory cytokines (IL‐1α, IFN‐γ, and IL‐6) levels between the Mtb‐infected TRF and control group (TRF‐TB and ALF‐TB) were similar. At 21 dpi, the TRF mice's bone marrow had a lower CD3^+^ and CD4^+^ T‐cell population and lower levels of CD4^+^ T cells in the lungs (Fig. 4H). Adaptive immune response characterized by infiltration of CD4^+^ T cells in the lungs is a key host immune response against Mtb, but a decrease in the number of CD4^+^ T cells did not compromise the containment of Mtb infection by the TRF mice. This suggests that TRF may enhance the function of CD4^+^ T cells. Our study is one of the first study to investigate the impact of TRF on Mtb infection, but an earlier report investigated the effect of CR on Mtb infection and showed that CR initiated before the Mtb infection resulted in a decreased number of CD3^+^CD4^+^ T cells in the lungs but not in the spleen of intravenously Mtb H37Rv‐infected DBA/2 mice at 40 dpi [26]. Differential effect of TRF and CR on tissue mycobacterial burden and immune cell distribution could be explained by differences in their energetics, in the mouse strain (C57BL/6 vs DBA/2), differences in timepoints (21 dpi vs 40 dpi) selected for sacrifice, and differences in the mode of infection (aerosol vs intravenous) [26].

A limitation of our study is that we did not assess how TRF affects the functionality of CD3^+^ or CD4^+^ T cells or the distribution of other immune cells (such as macrophages and neutrophils) involved in the host defense against Mtb. Also, the current study did not have a group in which TRF was continued throughout Mtb infection or initiated after Mtb infection. The inclusion of these groups and maintaining them for a longer period could help determine whether continued TRF or TRF initiated after Mtb infection might have a positive effect on the immune system. Age of the host might also influence the TRF‐induced changes observed in the metabolism and immune system.

In conclusion, our study demonstrated that TRF for 30 days remodels the host metabolic phenotype by altering amino acid and lipid metabolism, and these beneficial effects persist even after TRF is discontinued. However, discontinuing the TRF had no additional effect on the mycobacterial clearance at 21 dpi. Pre‐exposure to TRF affected both tissue‐level metabolism and the distribution of immune cells (CD3^+^ and CD4^+^ T cells) in Mtb‐infected mice. Since the decrease in the number of CD3^+^ and CD4^+^ T cells did not compromise the host's ability to clear mycobacteria, it indicates that pre‐exposure to TRF might improve the functionality of CD3^+^ and CD4^+^ T cells.

## Conflict of interest

The authors declare no conflict of interest.

## Author contributions

AG and RKN conceived and designed the experiments. RKN supervised the study. AG and NY performed experiments and analyzed data. AG and RKN wrote the manuscript. SD, RRKR and ARS helped in proteomics data acquisition and processing. NS and JSM helped in metabolomics data acquisition. RKN contributed to the funding.

## Supporting information


**Fig. S1.** Time‐restricted feeding (TRF) in C57BL/6 mice reduced body weight with a limited impact on glucose homeostasis.
**Fig. S2.** Time‐restricted feeding (TRF) in C57BL/6 mice induces metabolic remodeling in the liver.
**Fig. S3.** C57BL/6 mice aerosol infected with 100–400 colony‐forming units of *Mycobacterium tuberculosis* H37Rv show similar lung bacterial load and glucose homeostasis.
**Fig. S4.** Absolute number of CD3+ and CD3 + CD4+ T cells in (A) bone marrow, (B) lungs and (C) spleen at 21 dpi in the *Mycobacterium tuberculosis* H37Rv‐infected control (ALF) and experimental group (TRF).
**Fig. S5.** Time‐restricted feeding (TRF) induced changes in the liver metabolome that persisted post‐*Mycobacterium tuberculosis* H37Rv infection.
**Table S1.** List of deregulated serum metabolites in the time‐restricted feeding (TRF) mice post 30 days of TRF.
**Table S2.** List of deregulated liver metabolites in the time‐restricted feeding (TRF) mice post 30 days of TRF.
**Table S3.** List of the deregulated liver proteins in the time‐restricted feeding (TRF) mice post 30 days of TRF.
**Table S4.** List of deregulated serum metabolites in the time‐restricted feeding‐tuberculosis mice post 21 days of *Mycobacterium tuberculosis* H37Rv infection.
**Table S5.** List of deregulated liver metabolites in the time‐restricted feeding‐tuberculosis mice post 21 days *Mycobacterium tuberculosis* H37Rv infection.
**Table S6.** List of deregulated liver proteins in the time‐restricted feeding‐tuberculosis mice post 21 days *Mycobacterium tuberculosis* H37Rv infection.

## Data Availability

The data that support the findings of this study are available from the corresponding author (ranjan@icgeb.res.in) upon reasonable request. The data that support the findings of this study are openly available on Mendeley Data (doi:10.17632/ypnpckfx6z.1).
