# Supplementary material for: Time‐restricted feeding prior to Mycobacterium tuberculosis infection reduces tissue CD4+ T cells with limited impact on bacterial clearance
Source: FEBS Open Bio. 2026 Jun 4:10.1002/2211-5463.70263. Online ahead of print. doi: 10.1002/2211-5463.70263 (PMC13398685; doi:10.1002/2211-5463.70263)
Supplement: Supplementary file 1 — Fig. S1. Time‐restricted feeding (TRF) in C57BL/6 mice reduced body weight with a limited impact on glucose homeostasis. Fig. S2. Time‐restricted feeding (TRF) in C57BL/6 mice induces metabolic remodeling in the liver. Fig. S3. C57BL/6 mice aerosol infected with 100–400 colony‐forming units of Mycobacterium tuberculosis H37Rv show similar lung bacterial load and glucose homeostasis. Fig. S4. Absolute number of CD3+ and CD3 + CD4+ T cells in (A) bone marrow, (B) lungs and (C) spleen at 21 dpi in the Mycobacterium tuberculosis H37Rv‐infected control (ALF) and experimental group (TRF). Fig. S5. Time‐restricted feeding (TRF) induced changes in the liver metabolome that persisted post‐Mycobacterium tuberculosis H37Rv infection. Table S1. List of deregulated serum metabolites in the time‐restricted feeding (TRF) mice post 30 days of TRF. Table S2. List of deregulated liver metabolites in the time‐restricted feeding (TRF) mice post 30 days of TRF. Table S3. List of the deregulated liver proteins in the time‐restricted feeding (TRF) mice post 30 days of TRF. Table S4. List of deregulated serum metabolites in the time‐restricted feeding‐tuberculosis mice post 21 days of Mycobacterium tuberculosis H37Rv infection. Table S5. List of deregulated liver metabolites in the time‐restricted feeding‐tuberculosis mice post 21 days Mycobacterium tuberculosis H37Rv infection. Table S6. List of deregulated liver proteins in the time‐restricted feeding‐tuberculosis mice post 21 days Mycobacterium tuberculosis H37Rv infection. [file FEB4-9999-0-s001.pdf]

Time-restricted feeding prior to *Mycobacterium tuberculosis* infection reduces tissue CD4+ T-cells with limited impact on bacterial clearance

Ashish Gupta, Nidhi Yadav, Subhasmita Das, R. Rajendra Kumar Reddy, Nupur Sharma, Amol Ratnakar Suryawanshi, Jaswinder Singh Maras, Ranjan Kumar Nanda

Supplementary Figure S1

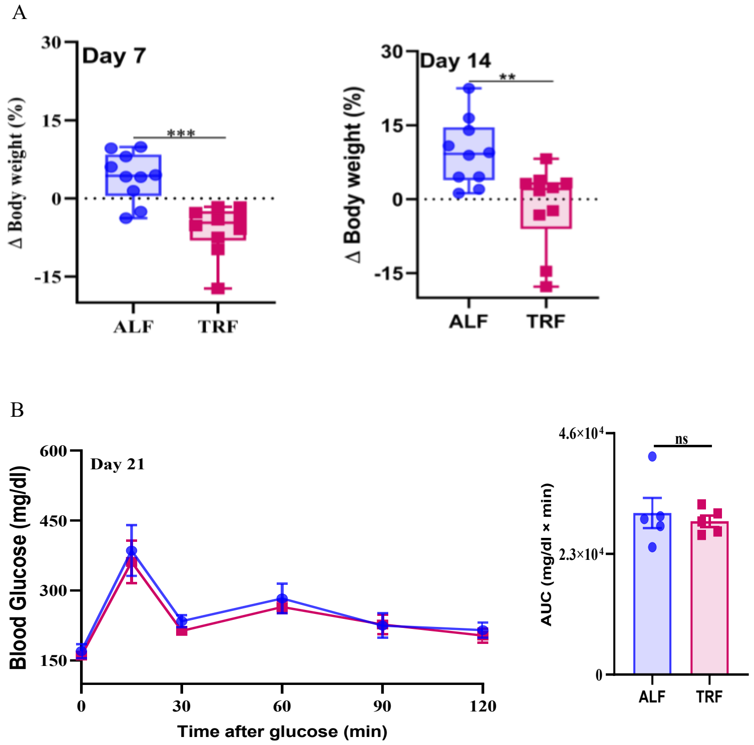

Supplementary Figure S1. Time-restricted feeding (TRF) in C57BL/6 mice reduced body weight with a limited impact on glucose homeostasis. A. Percentage body weight changes at Day 7 and 14 post-TRF. **B.** Intraperitoneal glucose tolerance test at Day 21 post-TRF. The area under the curve above the baseline is shown. Each dot represents a biological replicate. Statistical significance was determined using an unpaired t-test with Welch's correction in **A**. And a two-way repeated-measures ANOVA and Sidak's multiple comparisons tests for the intraperitoneal glucose tolerance test, and an unpaired t-test with Welch's correction for calculating the AUC in **B**. The error bars represent the data range, and the middle line in A represents the median. The error bar represents the standard error of the mean in **B.**

Supplementary Figure S2

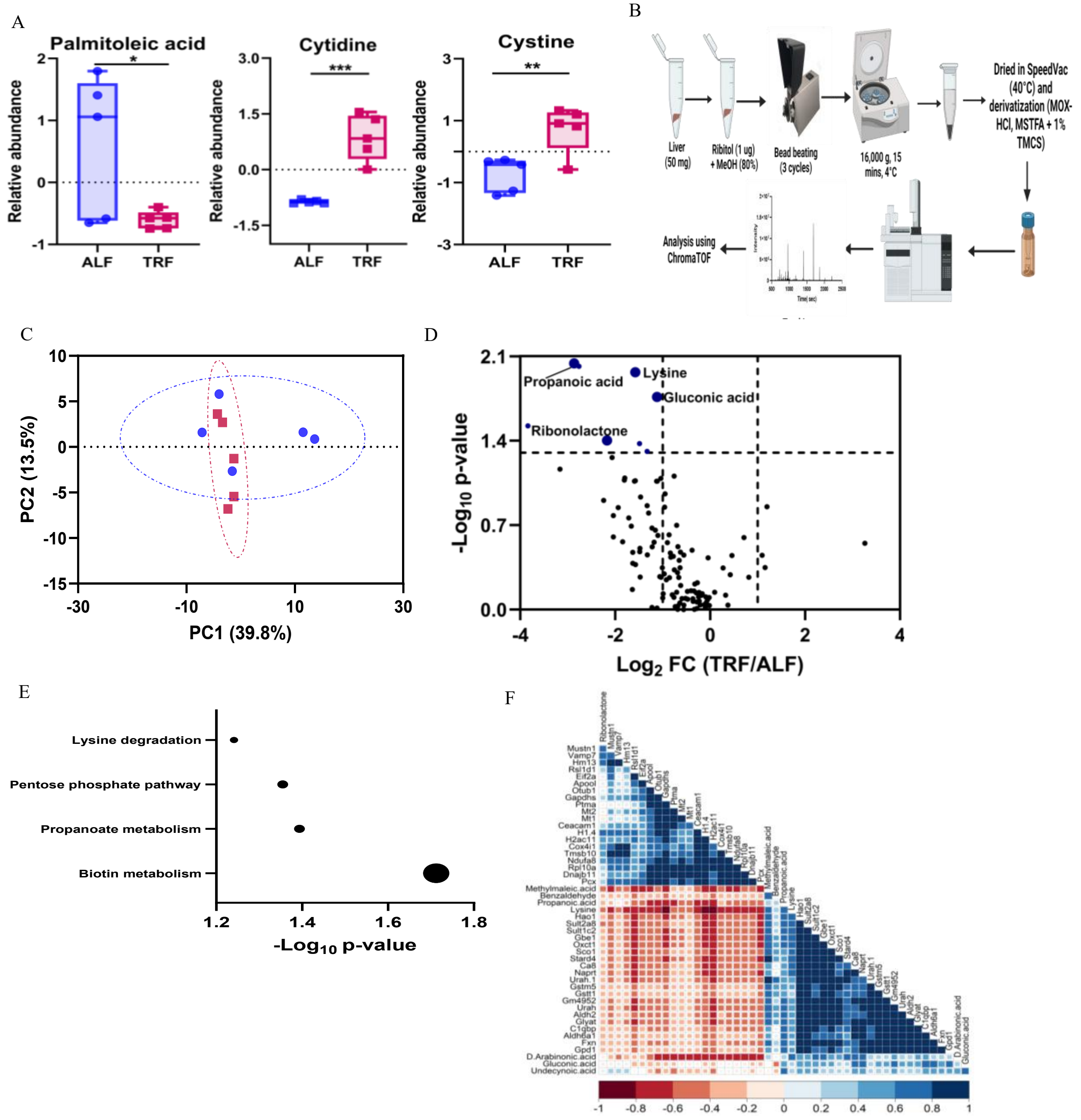

Supplementary Figure S2. Time-restricted feeding (TRF) in C57BL/6 mice induces metabolic remodelling in the liver. A. Relative abundance of palmitoleic acid, cytidine, and cystine in the serum measured by using gas chromatography mass spectrometry (GC-MS). **B.** Schematic representation of the method used for metabolite profiling from the TRF and ad libitum feeding (ALF) mice liver using GC-MS. **C.** Principal component analysis (PCA) of the liver metabolites between the TRF and ALF mice. **D.** Volcano plot showing the deregulated metabolites in the liver of the TRF and ALF mice. **E.** Metabolite set enrichment analysis (MSEA) of the deregulated metabolites. The dot size represents the enrichment ratio. **F.** Pairwise correlation of significantly deregulated (p-value ≤ 0.05; $\log_2$ Fold change ALF/TRF$\geq\pm1.0$) proteins and metabolites in the liver. Statistical significance was determined using an unpaired t-test with Welch's correction, and the error bar represents the range of the data, and the middle line represents the median in **A**. Each dot represents a biological replicate.

Supplementary Figure S3

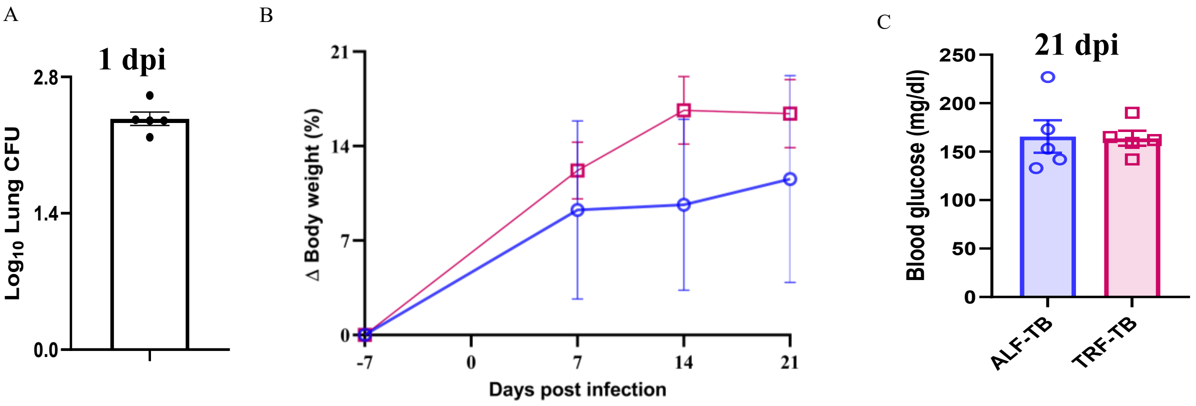

Supplementary Figure S3. **C57BL/6 mice aerosol infected with 100-400 colony-forming units of *Mycobacterium tuberculosis* H37Rv show similar lung bacterial load and glucose homeostasis. A.** Lung mycobacterial burden at 1 day post-infection (dpi). **B.** Percentage difference in bodyweight between the ad libitum feeding-tuberculosis (ALF-TB) and time-restricted feeding-TB (TRF-TB) mice. **C.** Random blood glucose levels at 21 dpi. Each dot represents a biological replicate. The error bar represents the standard error of the mean.

Supplementary Figure S4

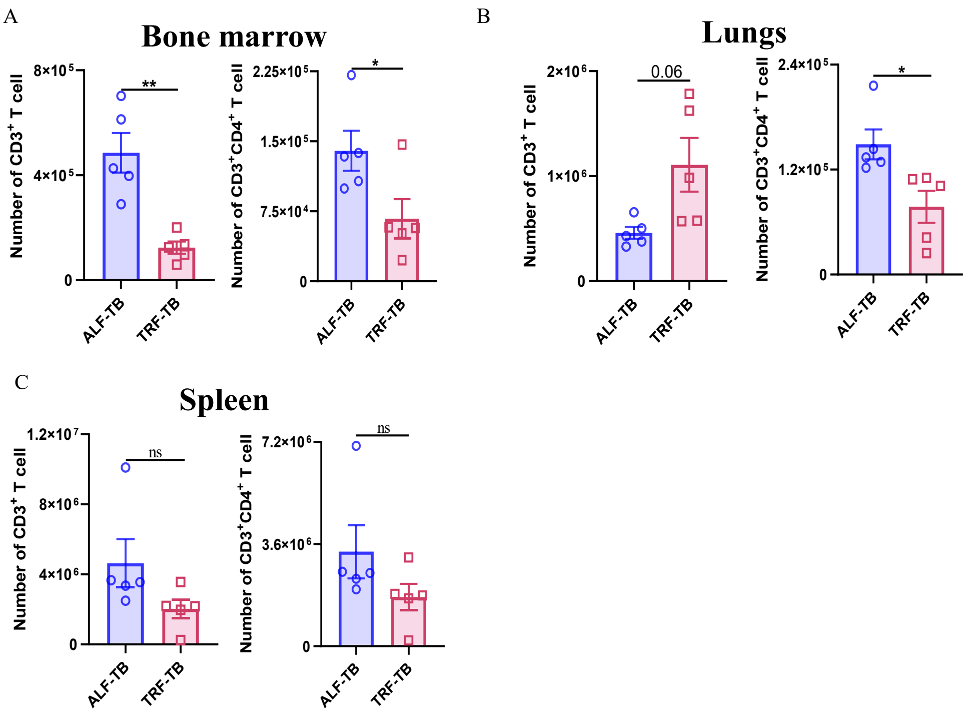

Supplementary Figure S4. **Absolute number of $CD3^+$ and $CD3^+CD4^+$ T cells in** (**A.**) bone marrow, (**B.**) lungs and (**C.**) spleen at 21 dpi in the *Mycobacterium tuberculosis* H37Rv-infected control (ad libitum feeding) and experimental group (time-restricted feeding). Each dot represents a biological replicate. The error bar represents the standard error of the mean. Statistical significance was determined using an unpaired t-test with Welch's correction. *p≤0.05 and **p≤0.01.

Supplementary Figure S5

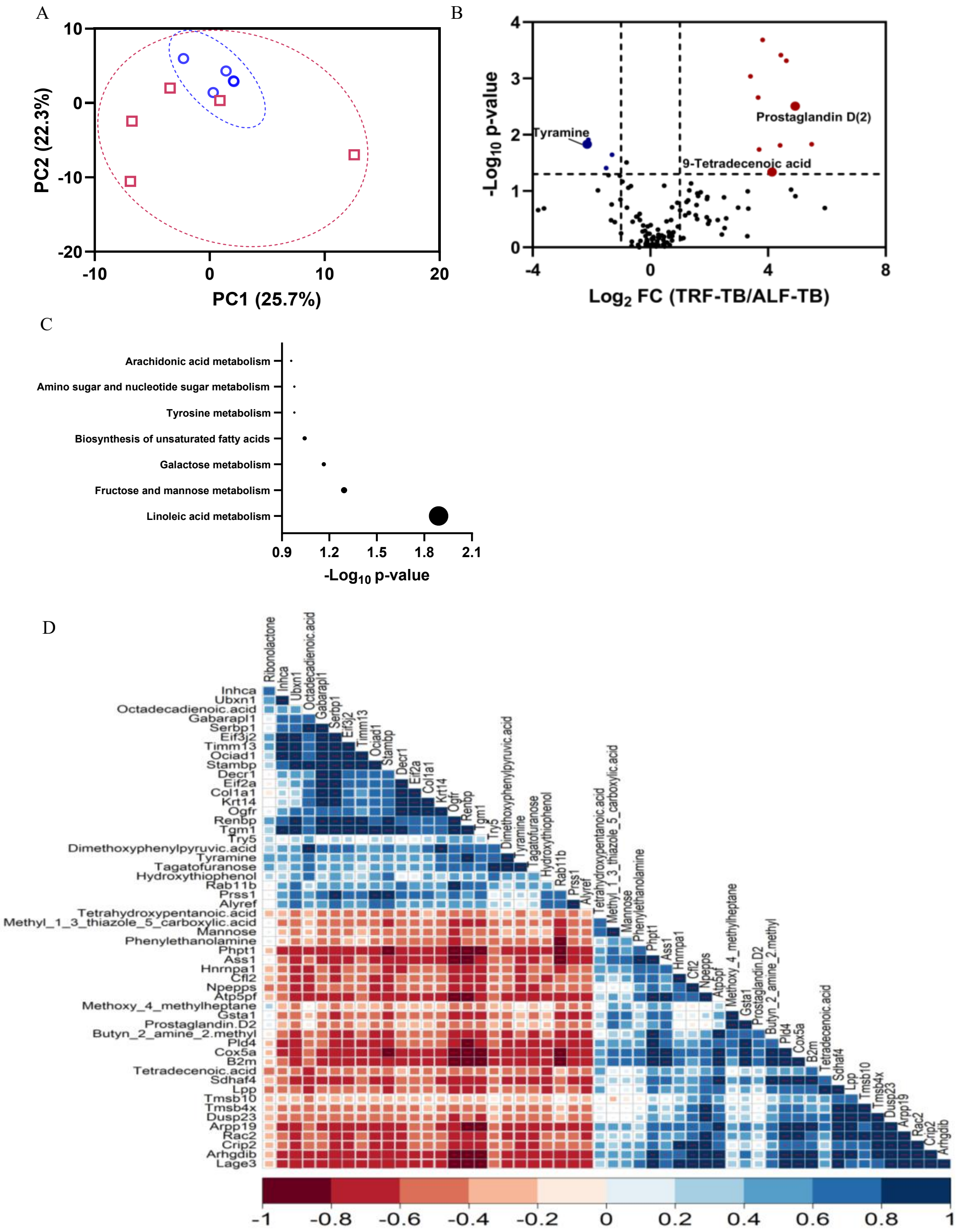

Supplementary Figure S5. Time-restricted feeding (TRF) induced changes in the liver metabolome that persisted post-*Mycobacterium tuberculosis* H37Rv infection. A. Principal component analysis (PCA) of the liver metabolites between the ad libitum feeding-tuberculosis (ALF-TB) and TRF-TB ALF mice. **B.** Volcano plot showing the deregulated metabolites in the liver of the ALF-TB and TRF-TB mice. **C.** Metabolite set enrichment analysis of the deregulated metabolites. The dot size represents the enrichment ratio. **D.** Pairwise correlation of significantly deregulated (p-value $\leq$ 0.05; $\log_2$ Fold change ALF-TB/TRF-TB$\geq\pm$1.0) proteins and metabolites in the liver.

Supplementary Table S1. List of deregulated serum metabolites in the time-restricted feeding (TRF) mice post 30 days of TRF.

Metabolic features	log_2FC (TRF/ALF)	p-value
Androstenedione	-3.16	0.010
Erabulenol B	-2.68	0.033
8-desoxy-19,20-epoxycytochalasin C	-2.56	0.012
Bacilotetrin B	-2.46	0.004
Fumagillin	-2.44	0.007
Dihydroepiheveadride	-2.37	<0.001
Maltophilin	-2.35	0.041
CerP(d18:1/16:0)	-2.31	0.002
8-Epiiridotrial glucoside	-2.28	0.038
Pestarhamnose B	-2.09	0.039
DG(17:2(9Z,12Z)/20:3(8Z,11Z,14Z)/0:0)[iso2]	-2.09	0.003
didesmethyl tocotrienol	-1.92	0.006
Asukamycin C-II	-1.65	0.034
(1-Ribosylimidazole)-4-acetate	-1.65	0.014
methyl 10-acetoxy-8,9-epoxy-2Z-decen-4,6-diynoate	-1.60	0.041
6-Hydroxyluteolin 6,3'-dimethyl ether 7,4'-disulfate	-1.56	0.026
N-propyl arachidonoyl amine	-1.49	0.043
Ptilosteroid B	-1.46	0.012
3_4-Dihydroxyphenylethyleneglycol	-1.46	0.049
CerP(d18:1/18:0)	-1.43	0.018
Asparacemosone D	-1.37	0.020
Dichloroacetic acid	-1.31	0.013
2_4.6-Trichloroanisole	-1.3	0.040
Streptomyceamide C	-1.19	0.048
Triangulyne E	-1.05	0.017
2,10-dimethyl 4-hydroxy-6-oxo-4-undecen-7-yne	-1.01	0.022
Farfugin A	-1.00	0.037
(2-acetamido-1-hydroxyethyl)phosphonic acid	1.05	0.048
Hexadecanedioic acid	1.10	0.018
Neuraminic acid	1.13	0.036
Acetonecyanohydrin	1.24	0.028
(6Z)-Octadecenoicacid	1.31	0.038
Hexadecanoic acid	1.32	0.018
Acrylamide	1.44	0.001
2-Hydroxy-2-methylbutyronitrile	1.69	0.005
6,9,12,15,18,21-Tetracosahexaynoic acid	1.76	0.046
Toluene	1.87	0.041
2-MethylpropanalO-methyloxime	1.89	0.038
1_4-Lactone	1.95	0.038
Phenolic steroid	2.06	0.018
Cytidine	2.20	0.016
2-methyl-2Z-hexenoic acid	2.31	0.023
Orthophosphate	2.36	0.041
Palmitoleic acid	2.42	0.043
Epopromycin B	2.42	0.048
(S)-1-Pyrroline-5-carboxylate	2.44	0.043
1alpha-fluoro-25-hydroxy-16,17,23,23,24,24-hexadehydrovitamin D3	2.57	0.025
1,3,6-Octatriene	2.67	0.049
PS(17:0/20:4(5Z,8Z,11Z,14Z))	2.86	0.019
4,6-dichloro-3,7-dihydroxy-1,9-dimethyldibenzofuran	2.96	0.045
12-Hydroxydodecanoicacid	3.68	0.024

Supplementary Table S2. List of deregulated liver metabolites in the time-restricted feeding (TRF) mice post 30 days of TRF.

Metabolic features	log_2FC (TRF/ALF)	p-value
Propanoic acid	-2.86	0.009
Methylmaleic acid	-2.76	0.01
Lysine	-1.57	0.01
D-Gluconic acid	-1.11	0.017
Benzaldehyde	-3.83	0.029
Ribonolactone	-2.17	0.039
D-Arabinonic acid	-1.48	0.042
10-Undecynoic acid	-1.32	0.048

Supplementary Table S3. List of the deregulated liver proteins in the time-restricted feeding (TRF) mice post 30 days of TRF.

Protein	Log_2 FC (TRF/ALF)	p-value	Protein	Log_2 FC (TRF/ALF)	p-value	Protein	Log_2 FC (TRF/ALF)	p-value
Hsd17b8	-2.99	0.007	Carnmt1	-4.88	0.010	Glyat	-5.31	<0.001
Gbe1	-6.63	<0.001	Casp6	-4.45	<0.001	Gm4952	-5.35	<0.001
Akr1e2	-2.70	0.004	Casp7	-2.41	0.013	Agl	-1.97	0.013
Hsd11b1	-3.20	0.009	Comt	-2.14	0.008	Pygb	-2.63	0.013
Ywhae	-3.92	0.015	Ctsd	-3.27	<0.001	Pygl	-3.60	0.002
Ywhaq	-1.51	0.008	Ctsf	-2.16	<0.001	Glod4	-1.87	0.006
Hpgd	-3.41	0.003	Ctsz	-2.30	0.002	Grhpr	-2.81	0.002
Plcg2	-2.44	<0.001	Alcam	-2.84	0.003	Grpel1	-2.53	0.009
Hao1	-6.53	<0.001	Clic1	-5.20	0.001	Gbp2b	-3.30	0.014
Akr1c14	-1.80	0.006	Clic4	-4.52	0.001	Guk1	-1.65	0.017
Hibch	-3.36	0.003	Cbx5	-2.37	0.006	Gbp2	-4.27	0.004
Acaa1a	-2.30	0.002	Cs	-3.49	0.011	H2-L	-2.18	0.005
Acaa1b	-2.27	0.001	Cd300lg	-1.01	0.003	H2-Q10	-1.93	0.014
Pcbd2	-1.56	0.001	Cfl1	-2.33	<0.001	H2-Aa	-2.32	0.008
Abat	-3.35	0.003	Cfl2	-2.73	0.001	Hspa1b	-1.94	0.006
Aldh9a1	-4.29	0.001	C1qbp	-6.44	0.003	Hspa4	-2.43	0.009
Pld4	-2.62	0.005	Cfd	-2.98	0.001	Hspa4l	-2.15	0.010
Urah	-5.35	<0.001	Cpne1	-1.32	0.017	Hmox1	-2.51	0.006
Oplah	-2.75	0.016	Ccs	-2.04	0.016	Hnrnpa1	-3.86	0.001
Hspd1	-3.42	0.004	Czib	-2.86	0.005	Hnrnpa3	-1.58	0.009
Acat2	-2.32	0.012	Smpdl3a	-2.16	0.009	Hnrnpa2b1	-1.97	0.001
Acat1	-2.00	0.002	Cdo1	-1.83	0.003	Hk3	-3.97	0.006
Acat3	-1.46	0.010	Csad	-3.96	<0.001	H6pd	-2.11	0.015
Acot12	-3.86	0.001	Cars1	-3.24	0.003	Hmgb1	-1.69	0.016
Actl6a	-1.91	0.006	Uqcrc1	-1.79	0.011	Hal	-4.75	0.001
Actr2	-4.81	0.002	Aco1	-3.33	0.005	Hgd	-1.99	0.012
Arpc1a	-4.47	0.004	Cndp2	-3.05	0.010	Hykk	-4.21	0.003
Arpc2	-4.08	0.003	Dazap1	-2.49	0.010	Hmgcs2	-2.05	0.015
Arpc3	-3.25	0.006	Bdh1	-2.85	0.006	Hyou1	-3.02	0.001
Arpc4	-4.48	0.001	Nit1	-3.72	0.002	Igha	-2.83	0.003
Arpc5	-3.12	0.009	Aldh4a1	-3.02	0.006	Iglc1	-2.08	0.011
Arpc1b	-3.34	0.006	Dguok	-1.36	0.013	Hpse2	-3.77	0.001
Actr3	-3.84	0.004	Dstn	-3.58	<0.001	Inmt	-5.27	<0.001
Apeh	-3.33	0.001	Dhfr	-3.62	0.003	Inhca	-4.22	<0.001
Acot7	-3.25	0.008	Dld	-2.99	0.001	Ide	-3.00	0.001
Acnat1	-3.75	<0.001	Dpys	-1.40	0.011	Itih2	-2.36	0.006
Nudt19	-5.24	0.003	Dmgdh	-4.75	0.002	Iscu	-2.66	0.003
Acsm3	-2.53	0.005	Dpp7	-2.86	0.004	Iah1	-4.43	0.001
Acsm5	-3.74	0.002	Dpp3	-1.47	0.002	Isoc2a	-3.05	0.008
Acot1	-2.68	0.012	Nudt4	-3.47	0.001	Isoc2b	-2.13	0.018
Acot8	-3.28	0.015	Dnajc1	-3.40	0.007	Idh3g	-3.00	0.005
Them4	-3.30	0.004	Eml2	-4.84	<0.001	Idh3b	-2.76	0.003
Crk	-2.55	0.017	Efhd2	-3.34	0.005	Kynu	-4.07	0.001
Hint2	-4.96	<0.001	Lage3	-1.34	0.015	Lancl2	-4.01	0.002
Nudt9	-1.84	0.013	Eef2	-4.42	0.001	Lta4h	-2.53	0.005
Arf1	-1.81	0.002	Elob	-3.23	0.004	Gulo	-2.76	<0.001
Arf3	-1.42	0.005	Erap1	-5.30	0.001	Mesd	-2.61	0.002
Arf4	-2.55	0.007	Erp44	-2.67	0.015	Gba1	-3.96	0.001
Arf6	-1.63	0.011	Lactb2	-1.74	0.002	Lipa	-3.19	0.004
Nudt5	-2.89	0.008	Mecr	-3.05	0.015	Mup1	-2.76	0.005
Gpt	-3.83	0.003	Echdc2	-3.44	0.003	Mup22	-2.46	0.005
Aldh1a1	-2.99	0.004	Egfr	-1.50	0.012	Mup13	-2.96	0.002
Aldh1a7	-2.64	0.009	Ero1a	-2.53	0.010	Mup14	-3.42	0.012
Aldh2	-6.08	0.001	Akr1c6	-2.13	0.005	Mup20	-2.82	<0.001
Aox2	-2.33	0.013	Pcyt2	-4.16	<0.001	Mup21	-3.90	0.001
Aox3	-2.01	0.003	Eif4ebp2	-1.61	0.002	Mup3	-1.53	0.016
Aox1	-2.15	0.002	Ezr	-2.82	0.002	Mup7	-2.66	0.007
Akr1c12	-2.34	0.008	Capza1	-3.76	0.001	Gmppb	-2.41	0.002
Akr1a1	-1.06	0.016	Capza2	-4.63	<0.001	Gmppa	-5.01	<0.001
Akr1b1	-2.55	0.006	Capzb	-3.72	0.001	Mpi	-2.87	0.004
Akr1d1	-2.65	0.005	Fdps	-3.00	0.008	Acsf2	-4.38	0.009
Akr1b8	-3.21	0.002	Fscn1	-3.45	0.009	Acadm	-2.61	0.009
Aifl	-4.75	<0.001	Fabp7	-3.61	0.001	Mat2b	-2.13	0.011
Serpina1d	-2.96	0.003	Fcgr2b	-4.56	<0.001	Metap1d	-3.49	0.003
Ttpa	-4.42	0.009	Fdx2	-2.32	0.006	Aldh6a1	-5.44	0.002
Anxa1	-3.79	<0.001	Cp	-2.63	0.001	Mettl27	-2.01	0.008
Anxa2	-2.05	0.017	Fggy	-3.92	0.006	Mttp	-4.33	0.001
Anxa6	-1.65	0.001	Fgg	-2.59	0.011	Map1lc3b	-3.34	0.003
Aifm1	-2.97	0.004	Blvrb	-2.53	0.002	Mien1	-3.71	0.001
Rars1	-4.01	0.001	Fxn	-5.78	0.001	Nudt8	-2.94	0.008
Armc1	-3.78	0.002	Aldoa	-1.76	0.015	Pmpca	-3.16	0.005
Nat2	-2.48	<0.001	Aldob	-1.84	0.014	Mapk14	-5.22	0.013
Dnpep	-3.06	<0.001	Aldoc	-1.19	0.015	Msn	-1.07	0.014
Atp5f1b	-3.68	<0.001	Fh	-3.66	0.016	Mocs2	-1.73	0.013
Atp5f1d	-1.97	0.006	Galm	-2.43	0.011	Osgep	-4.13	0.013
Abce1	-1.93	0.008	Galt	-2.18	0.016	Nherf1	-1.44	0.004
Acly	-3.06	0.003	Lgals1	-2.12	0.014	Nagk	-4.22	0.012
Pfkl	-3.17	0.004	Lgals3	-4.42	0.001	Acyl	-1.17	0.005
Pfkm	-4.32	0.001	Lgals3bp	-1.90	0.001	Nadk2	-2.19	0.009
Papss2	-3.24	0.006	Gabarapl2	-3.12	0.001	Nadk2	-1.41	0.018
Ephx2	-4.53	0.001	Ifi30	-2.33	0.007	Ndufa2	-3.65	0.009
Atic	-1.96	0.010	Gsn	-2.48	0.005	Cyb5r3	-2.08	0.011
Baat	-3.24	0.005	G6pdx	-3.92	0.003	Me1	-2.58	0.006
Blvra	-2.93	0.007	Gatd1	-2.32	0.008	Asah2	-2.47	<0.001
Nudt2	-3.60	0.004	Gfpt1	-2.05	0.016	Nampt	-3.56	0.015
Calm2	-1.66	<0.001	Glrx5	-3.21	0.003	Naprt	-6.06	<0.001
Calr	-4.32	0.003	Gcdh	-4.19	0.015	Qprt	-5.10	<0.001
Calu	-3.96	<0.001	Gsta3	-1.53	0.012	Nolc1	-2.27	0.013
Ca3	-2.08	0.013	Gstm1	-3.83	0.003	Nit2	-1.52	0.012
Ca8	-7.32	<0.001	Gstm3	-4.48	<0.001	Ogfr	-1.60	0.013
Cbr3	-2.46	0.006	Gstm4	-4.43	0.010	Oxnad1	-3.08	0.015
Ces1d	-3.72	0.003	Gstm5	-7.00	0.004	Pank3	-2.71	0.006
Ces1f	-3.11	0.004	Gstm7	-2.69	0.010	Park7	-2.93	0.001
Ces3a	-4.46	0.004	Gstt1	-6.47	0.005	Ppib	-1.03	0.008
Ces3b	-5.24	0.001	Gstm1	-3.47	0.004	Ppic	-1.81	0.004
Ces2f	-2.75	0.001	Gstp1	-4.93	<0.001	Ppid	-3.20	0.002
Cmbl	-1.74	0.010	Gk	-2.21	0.004	Fkbp4	-4.45	0.005
Cpb2	-1.83	0.013	Gpd1	-5.68	0.012	Prdx1	-2.39	0.010
Ctsa	-1.42	0.004	Hsd17b4	-1.73	<0.001	Crot	-2.18	0.009

Protein	Log_2 FC (TRF/ALF)	p-value	Protein	Log_2 FC (TRF/ALF)	p-value	Protein	Log_2 FC (TRF/ALF)	p-value
Prdx4	-1.57	0.002	Sult2a8	-6.41	<0.001	Hadh	1.68	0.003
Prdx5	-4.59	0.001	Sod2	-1.92	0.014	Hagh	2.21	0.013
Acox1	-2.64	0.004	Tax1bp3	-2.76	<0.001	Impa1	1.73	0.001
Crot	-2.18	0.009	Txndc12	-3.73	0.006	Asrgl1	1.38	<0.001
Nudt7	-3.44	0.002	Txnrd1	-2.75	0.003	Idi1	1.80	0.001
Hsd17b4	-1.73	<0.001	Txnrd3	-1.61	0.001	F11r	3.18	0.007
Pipox	-1.85	0.003	Txn2	-2.90	0.004	Krt18	3.13	0.007
Pex7	-2.69	<0.001	Tstd3	-3.37	0.007	Krt8	2.10	0.004
Pctp	-1.82	0.009	Taco1	-2.84	0.010	Glo1	1.29	0.013
Pitpna	-2.16	0.006	Tango2	-2.28	0.003	Rpl14	2.83	0.006
Pitpnb	-1.68	0.006	Urah	-5.33	<0.001	Rpl12	3.18	0.002
Pck1	-3.74	0.005	Tpi1	-2.13	0.008	Lrrc59	2.55	0.017
Pmm2	-1.87	0.012	Tpm3-rs7	-3.27	0.010	Fuom	1.89	0.003
Lcp1	-4.08	0.001	Lsm4	-1.77	0.015	Acp1	1.97	<0.001
Pls3	-2.38	0.003	Ubqln1	-2.40	0.009	Man2b1	1.43	0.006
Plxnb2	-2.72	0.005	Ube2n	-2.85	<0.001	Mif	1.55	0.002
Pzp	-3.89	0.002	Ube2i	-2.26	0.015	Mdp1	1.70	0.011
Pfn1	-2.34	0.008	Ubxn1	-2.45	0.001	Mlec	1.43	0.018
Pfn2	-3.04	<0.001	Cmpk1	-4.32	0.002	Mt1	3.97	0.014
Psap	-3.66	0.004		-1.70	0.016	Mt2	3.85	0.010
Ptgr2	-2.83	<0.001	Bles03	-3.54	0.001	Mars1	1.93	0.009
Ptgr3	-3.20	0.004	Nudt14	-2.00	0.010	Mia3	1.37	0.014
Prss1	-1.40	0.007	Uroc1	-1.23	0.001	Apool	3.44	0.007
Psme1	-2.69	0.001	Ugp2	-4.06	0.013	Immt	1.86	0.011
Psme2	-4.54	<0.001	Rad23a	-1.36	0.008	Hm13	3.77	0.016
Psma1	-2.85	0.004	Bphl	-5.10	0.006	Mustn1	4.06	0.009
Psma2	-3.58	0.001	Vasp	-4.45	<0.001	Galns	1.01	0.008
Psma3	-2.58	0.002	Vwa5a	-3.02	0.012	Gns	1.57	0.008
Psma4	-2.63	0.002	Wdr1	-3.21	0.004	Ndufa8	5.26	<0.001
Psma5	-3.49	0.002	Xpnpep1	-2.93	0.017	Ndufb10	3.20	0.008
Psma6	-3.92	0.001	Azgp1	-3.01	0.001	Ndufv3	1.33	0.012
Psma7	-2.59	0.001	Decr1	2.49	<0.001	Lcn2	2.68	0.004
Psmb9	-4.70	<0.001	Psmd3	2.86	<0.001	Nhlrc3	1.39	<0.001
Psmb1	-3.86	0.002	Pdap1	1.90	<0.001	Ddx21	1.47	0.013
Psmb10	-2.91	0.002	Mthfsl	1.59	0.009	Rexo2	1.08	0.003
Psmb2	-3.53	<0.001	Mthfs	1.34	0.008	Fahd1	1.54	0.002
Psmb3	-3.44	0.003	Dbi	1.43	0.007	Psip1	1.60	0.017
Psmb4	-2.75	0.001	Acyp1	1.08	0.014	Ppif	1.65	0.002
Psmb5	-2.53	0.015	Adss2	1.31	0.014	Decr2	2.61	0.009
Psmb6	-1.62	0.005	Aamdc	2.50	<0.001	Ethe1	1.93	0.003
Psmb7	-3.12	<0.001	Orm1	2.51	0.001	Pgam1	1.31	0.004
Psmb8	-3.33	0.005	Gla	1.01	0.010	Pir	1.07	0.015
P4hb	-5.14	0.001	Kgd4	1.60	0.011	Pqbp1	3.35	0.007
Fam136a	-1.93	0.016	Arsb	1.18	0.016	Polr2m	2.33	0.016
Ppm1a	-3.26	0.001	Arsg	3.19	0.004	Pawr	2.08	0.002
Ppm1b	-3.73	0.006	Got1	1.34	0.001	Ctsl	1.73	0.001
Ptprk	-2.37	0.004	Aspdh	1.01	0.009	Mix23	1.70	0.010
F2	-3.19	0.009	Atp5f1a	2.25	0.014	Ptma	4.31	0.006
Pnp	-2.77	0.013	Naxd	1.62	0.002	Pnpo	1.78	<0.001
Chac2	-3.80	0.003	Epb41l2	2.77	0.005	Pcx	3.96	0.001
Ces2a	-3.29	0.001	Glb1	1.05	0.015	Rab1a	1.43	<0.001
Ces2e	-2.59	0.011	Gusb	1.08	0.007	Rab11b	2.16	0.001
Plpbp	-1.22	0.017	Fhit	1.74	0.001	Rab5b	1.86	<0.001
Pklr	-2.25	0.011	Znf207	1.32	0.004	Rab5c	1.62	0.001
Cryz	-4.40	<0.001	Ceacam1	3.49	0.001	Rab7a	1.62	<0.001
Lamtor5	-1.93	0.016	Clybl	1.14	0.003	Ralb	1.22	<0.001
Ranbp1	-1.31	0.007	Ccdc12	2.22	0.012	Rap1b	1.73	0.005
Rras2	-1.95	0.008	Ccdc25	1.00	0.004	Rgn	1.53	0.002
Pde6d	-2.43	<0.001	Ncapd3	2.97	0.008	Rhog	1.49	0.016
Rbks	-2.15	0.017	Mmab	1.56	<0.001	Rsl1d1	4.03	<0.001
Rnase4	-1.93	0.009	Crp	1.81	0.001	Rpl10a	3.79	0.003
Alkbh5	-1.77	0.005	Csrp1	1.74	0.018	Spr	1.51	0.003
Sco1	-7.27	<0.001	Csrp2	2.96	<0.001	Septin9	1.32	0.008
Sec14l2	-2.91	0.007	Cda	2.54	<0.001	Cpped1	1.42	<0.001
Sephs1	-2.48	0.010	Cox4i1	5.21	0.003	Srp68	2.74	0.007
Sephs2	-1.90	0.002	Dync1li1	2.27	0.007	Rps10	3.39	0.012
Selenbp2	-1.58	0.014	Dap	1.89	0.001	Mrps31	1.80	0.015
Septin7	-3.52	0.007	Top1	1.57	0.012	Rack1	2.29	0.002
Serpina3k	-2.24	0.003	Polr2a	2.38	0.013	Slc38a3	2.39	0.014
Shmt1	-1.74	0.011	Dnajb12	1.95	<0.001	Sord	1.56	0.006
Ppp2ca	-2.27	0.001	Dnajb11	5.20	0.001	Sptbn1	3.41	<0.001
Ppp2cb	-2.62	0.003	Rpn1	3.37	<0.001	Sf3b2	2.07	0.006
Ppp5c	-3.01	0.002	Eif2a	3.54	0.003	Sfpq	2.07	<0.001
Ppp1ca	-3.70	0.003	Eif3a	1.99	<0.001	Sod1	1.66	0.012
Ppp1cb	-2.31	0.001	Eif4g1	2.81	0.005	Tmsb10	5.50	<0.001
Tf	-2.15	0.001	Eif4g2	1.94	0.012	Tmsb4x	3.32	0.001
Serpinb6	-2.67	0.005	Ewsr1	1.84	0.002	Tor1aip1	2.71	0.002
Sh3bgrl3	-1.76	0.013	Fam107b	2.64	<0.001	Purb	2.72	0.005
Sh3bgrl2	-2.27	0.001	Fbp1	2.08	<0.001	Vcp	3.16	0.008
Acads	-1.07	0.017	Gnpda1	1.08	0.005	Otub1	5.73	<0.001
Sar1a	-2.03	0.013	Gnpda2	1.60	0.001	Ubap2l	1.87	0.008
Abhd11	-3.73	0.009	Glrx2	3.18	0.008	Acadvl	2.06	0.011
Atp1a1	-2.10	0.009	Gpx1	2.12	0.001	Vamp7	4.67	0.015
Atp1b1	-2.30	0.004	Gss	1.61	<0.001	Pepd	1.53	0.002
Slco1a1	-2.85	0.002	Gapdh	2.47	0.003	Prdx2	-2.15	0.007
Sri	-4.40	0.015	Gapdhrt	1.93	0.002	Nudt7	-3.44	0.002
Ddx39b	-3.72	0.005	Gapdhs	4.27	<0.001	Pmm2	-1.87	0.012
Spryd4	-2.55	0.016	Gyg1	1.16	0.006	Lcp1	-4.08	0.001
Stard10	-2.46	0.007	Gchfr	1.20	0.003	Pls3	-2.38	0.003
Stard4	-5.57	<0.001	Hras	1.51	<0.001	Plxnb2	-2.72	0.005
Stard10	-1.96	0.016	Kras	2.58	0.008	Gpd1l	-3.44	0.007
Scp2	-3.00	0.001	Nras	1.67	0.002	Pex7	-2.69	<0.001
Hspa9	-1.86	0.005	Ran	1.57	0.002	Pctp	-1.82	0.009
Sdha	-1.57	0.002	Sgsh	2.88	0.006	Pitpna	-2.16	0.006
Suclg1	-3.24	<0.001	H1-3	3.10	0.007	Pitpnb	-1.68	0.006
Sucla2	-2.94	0.004	H1-4	5.86	<0.001	Pck1	-3.74	0.005
Suclg2	-2.60	0.005	H2ac11	4.16	0.001	Crot	-2.18	0.009
Oxct1	-5.40	<0.001	H2bc23	2.73	0.006	Hsd17b4	-1.73	<0.001
Sult1c2	-5.34	0.002	Adhfe1	1.89	0.010	Pipox	-1.85	0.003
Prdx5	-4.59	0.001	Acox1	-2.64	0.004	Pipox	-1.85	0.003

Supplementary Table S4. List of deregulated serum metabolites in the time-restricted feeding-tuberculosis (TRF-TB) mice post 21 days of *Mycobacterium tuberculosis* H37Rv infection compared to the ad libitum control group (ALF-TB).

**Metabolic features**	**log_2 FC (TRF-TB/ALF-TB)**	**p-value**
3-(Uracil-1-yl)-L-alanine	-1.58	<0.001
Benzyl(2R_3S)-2-methyl-3-hydroxybutanoate	-2.04	0.030
PI(O-20:0/20:0)	-2.09	0.042
PS(17:1(9Z)/22:6(4Z,7Z,10Z,13Z,16Z,19Z))	-1.87	0.041
Vitamin D3	1.02	0.011
[FA(6:0)]3Z-hexenol	1.82	0.050
16a-Hydroxyestrone	1.39	0.011
2-Methylpropanaloxime	1.33	<0.001
7-hydroxy-10E,16-heptadecadien-8-ynoic acid	1.09	0.004
Annopurpuricin E	1.70	0.016
Botrydial	3.35	0.029
CerP(d18:1/16:0)	1.41	0.035
CerP(d18:1/18:0)	1.47	0.021
CerP(d18:1/20:0)	1.45	0.002
Enteromycin	1.30	0.045
Marinacarboline A	1.44	0.031
methyl (Z)-3-(3,4-dihydroxyphenyl)-2-formamidoacrylate	1.57	0.047
N-Ribosylnicotinamide	1.58	0.005
PA(12:0/0:0)	1.50	0.010
PC(17:2(9Z,12Z)/0:0)	1.79	0.032
PE(18:3(6Z,9Z,12Z)/0:0)	2.57	0.008
Pukeleimide A	1.43	0.028

Supplementary Table S5. List of deregulated liver metabolites in the time-restricted feeding-tuberculosis (TRF-TB) mice post 21 days *Mycobacterium tuberculosis* H37Rv infection.

**Metabolic features**	**$\log_2$ FC (TRF-TB/ALF-TB)**	**p-value**
2,3-Dimethoxyphenylpyruvic acid	-2.11	0.012
Tyramine	-2.15	0.015
4-Hydroxythiophenol	-1.50	0.039
Tagatofuranose	-1.30	0.023
2,3,4,5-Tetrahydroxypentanoic acid-1,4-lactone, tris(trimethylsilyl)-	5.48	0.015
3-Butyn-2-amine, 2-methyl-	3.66	0.002
3-Methoxy-4-methylheptane	3.69	0.018
4-Methyl-1,3-thiazole-5-carboxylic acid	3.82	<0.001
9,12-Octadecadienoic acid	4.41	0.015
9-Tetradecenoic acid	4.13	0.046
Ribonolactone	4.44	<0.001
Mannose	4.62	<0.001
Phenylethanolamine	3.40	0.001
Prostaglandin D(2)	4.92	0.003

Supplementary Table S6. List of deregulated liver proteins in the time-restricted feeding-tuberculosis mice (TRF-TB) post 21 days *Mycobacterium tuberculosis* H37Rv infection.

Protein	$\log_2$ FC (TRF-TB/ALF-TB)	p-value
Decr1	-2.07	0.002
Col1a1	-3.49	<0.001
Eif2a	-4.49	<0.001
Eif3j2	-3.93	<0.001
Gabarapl1	-2.81	<0.001
Inhca	-3.24	0.002
Krt14	-3.39	<0.001
Timm13	-4.01	<0.001
Renbp	-3.64	<0.001
Ociad1	-1.78	0.001
Ogfr	-4.10	<0.001
Prss1	-2.33	0.001
Tgm1	-5.09	<0.001
Rab11b	-1.38	0.001
Serbp1	-2.36	<0.001
Stambp	-3.14	<0.001
Alyref	-1.82	0.002
Try5	-3.34	0.001
Ubxn1	-2.97	<0.001
Phpt1	2.68	<0.001
Akr1c14	1.57	<0.001
Pld4	3.55	<0.001
Dbi	1.28	0.002
Lrpap1	1.53	<0.001
Ass1	2.27	0.001
Atp5pf	2.56	<0.001
B2m	3.21	0.001
Arpp19	3.59	0.001
Cbr1	1.91	0.001
Ctss	1.07	0.001
Cfl2	3.49	0.002
Ckb	2.18	<0.001
Cstb	1.22	0.001
Crip2	3.76	0.001
Cox5a	3.80	0.001
Dusp23	2.35	0.001
Dynlrb1	1.81	<0.001
Lage3	3.07	<0.001
Fabp2	1.44	<0.001
Gsta1	3.49	0.001
Hnrnpa1	3.60	0.002
Hmgb2	2.19	<0.001
Lpp	3.45	0.002
Me1	1.17	<0.001
S100a10	2.25	0.001
Npepps	3.47	0.001
Rac1	1.82	<0.001
Rac2	3.51	<0.001
Arhgdib	3.51	<0.001
Rfk	2.20	0.001
2200002D01Rik	3.44	0.002
Sdhaf4	4.94	<0.001
Tmsb10	6.78	0.002
Tmsb4x	5.29	0.001
Ufc1	1.84	<0.001
Nedd8	1.50	<0.001
